# Alcohol reduces muscle fatigue through atomistic interactions with nicotinic receptors

**DOI:** 10.1038/s42003-018-0157-9

**Published:** 2018-10-03

**Authors:** Hamid R. Noori, Christian Mücksch, Valentina Vengeliene, Kai Schönig, Tatiane T. Takahashi, Nuriya Mukhtasimova, Maryam Bagher Oskouei, Matias Mosqueira, Dusan Bartsch, Rainer Fink, Herbert M. Urbassek, Rainer Spanagel, Steven M. Sine

**Affiliations:** 1 Institute of Psychopharmacology, Central Institute of Mental Health, Medical Faculty Mannheim, Heidelberg University, J5, 68159 Mannheim, Germany; 2 Neuronal Convergence Group, Max Planck Institute for Biological Cybernetics, Max Panck Ring 8, 72076 Tübingen, Germany; 3 Physics Department and Research Center OPTIMAS, University of Kaiserslautern, Erwin-Schrödinger Strasse 46, 67663 Kaiserslautern, Germany; 4 Courant Institute for Mathematical Sciences, New York University, 251 Mercer Street, New York, NY 10012 USA; 5 Department of Molecular Biology, Central Institute of Mental Health, Medical Faculty Mannheim, Heidelberg University, J5, 68159 Mannheim, Germany; 6 Receptor Biology Laboratory, Department of Physiology and Biomedical Engineering, Mayo Clinic College of Medicine, 200 First St. SW, Rochester, MN 55905 USA; 7 Institute of Physiology and Pathophysiology, Heidelberg University, Im Neuenheimer Feld 326, 69120 Heidelberg, Germany; 8 Present Address: Neuronal Convergence Group, Max Planck Institute for Biological Cybernetics, Max Planck Ring 8, 72076 Tübingen, Germany

## Abstract

Alcohol consumption affects many organs and tissues, including skeletal muscle. However, the molecular mechanism of ethanol action on skeletal muscle remains unclear. Here, using molecular dynamics simulations and single channel recordings, we show that ethanol interacts with a negatively charged amino acid within an extracellular region of the neuromuscular nicotinic acetylcholine receptor (nAChR), thereby altering its global conformation and reducing the single channel current amplitude. Charge reversal of the negatively charged amino acid abolishes the nAChR-ethanol interaction. Moreover, using transgenic animals harboring the charge-reversal mutation, ex vivo measurements of muscle force production show that ethanol counters fatigue in wild type but not homozygous αE83K mutant animals. In accord, in vivo studies of motor coordination following ethanol administration reveal an approximately twofold improvement for wild type compared to homozygous mutant animals. Together, the converging results from molecular to animal studies suggest that ethanol counters muscle fatigue through its interaction with neuromuscular nAChRs.

## Abstract (web summary)

Hamid Noori et al. show that ethanol reduces muscle fatigue through its interaction with a negatively charged residue on the extracellular side of the nicotinic acetylcholine receptor. This work provides molecular insights on the ethanol effects on the peripheral nervous system.

## Introduction

Ethanol interacts with a variety of molecular targets within the central and peripheral nervous systems, including the nicotinic acetylcholine receptor (nAChR)^1–6^. Electrophysiological studies of neuronal α2β4, α4β4, α2β2, and α4β2 nAChR subtypes predominantly suggest that ethanol interacts within the hydrophobic transmembrane domains of the nAChR^7–9^. However, the exact molecular mechanisms of ethanol action and the consequent conformational changes of nAChR remain largely unknown. Furthermore, it is well-known that ethanol, as an intoxicating agent that can lead to abuse and dependence, also causes motor impairment in human and laboratory animals^1^. Many organs and tissues, including skeletal muscle^10–13^, are affected by acute alcohol intoxication and by chronic consumption. Yet, due to the dominant focus of alcohol research on brain and liver function, few studies have been conducted to investigate the impact of acute ethanol intoxication on skeletal muscle^14–16^. Acute exposure to alcohol interferes with excitation–contraction coupling in skeletal muscle^17–19^, muscle metabolism^2^, muscle performance, and recovery^11,12^. In the majority of drinkers, chronic consumption results in alcoholic skeletal myopathy^13^. Nonetheless, the potential relevance of the neuromuscular junction for ethanol-induced effects, particularly the nAChR, remains unclear.

To address this question, we apply a multi-disciplinary approach. We perform molecular dynamics simulations on the relaxed, energy minimized cryo-electron microscopic structure of neuromuscular nAChR^20^ to identify sites of interaction with ethanol, as well as conformational changes associated with ethanol interaction. We then use in silico mutagenesis to alter the interacting amino acid and the surrounding cavity to design a nAChR with reduced sensitivity to ethanol. Following the in silico studies, we conduct single channel recordings of wild type and mutant nAChRs to evaluate the computational prediction of reduced ethanol sensitivity. Based on the successful validation of the predictions, we utilize CRISPR-CAS9 to generate a new transgenic line of rats carrying the identified mutation. Ex vivo measurements of muscle fatigue, as well as in vivo testing of motor, coordination and muscle strength, in wild type and transgenic rats, confirm the multi-scale impact of the ethanol interactions with nAChR at molecular level on muscle function in living organism.

## Results

### Molecular dynamics simulations

**Fig. 1:**
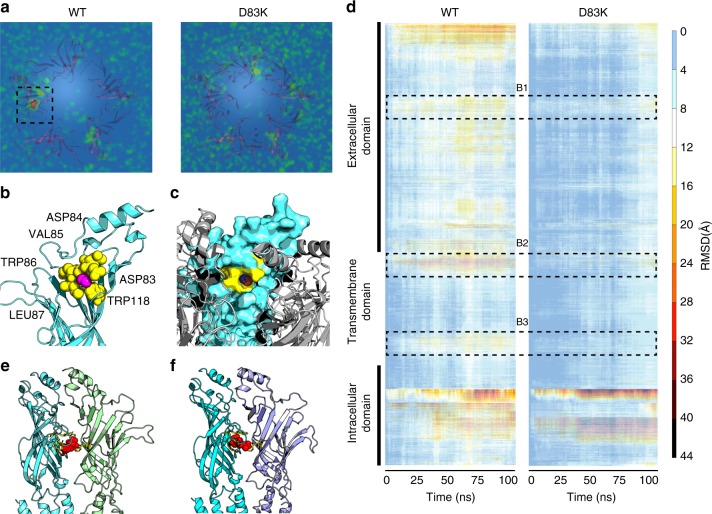
Molecular dynamics simulations reveals site of action of ethanol at extracellular domain. MD simulations suggest that ethanol occupies an area in the vicinity of α83 and induces fluctuations in nAChR conformation that are prevented by the D83K mutation of neuromuscular nAChR. **a** Density distribution (averaged over 100 ns) of ethanol molecules at 25 mM concentration. The selected area (extracellular domain of the α-subunit) shows the space with the highest density (in comparison to other receptor areas such as TMD) of ethanol during the MD simulation. In contrast, the average density distribution of ethanol molecules surrounding the αD83K receptor shows a homogeneous distribution of the molecules within the simulation space, suggesting ethanol is not attracted toward the mutated receptor. **b** Ethanol molecules interact with a cavity α83–87 (yellow) in the extracellular domain of the α-subunit (light blue). Spherical presentation of the cavity shows αD83, D84, V85, W86, L87 (yellow), and W118 that forms the cavity-base for ethanol molecule (pink). **c** Accessibility of the cavity by ethanol molecules is shown by surface presentation of the α-subunit. **d** All-residue RMSD of the backbone structure of WT and D83K receptors further supports the lack of ethanol-induced conformational fluctuations of the D83K receptor in the presence of ethanol. The area B1 relates to amino acids α65–88 that is associated with the accumulation of ethanol particles as in subfigure 1. The area B2 is the interface area between the ECD and TMD comprised by α188–208. B3 is the TMD area associated with the hydrophobic girdle (αL251, S252 and V255, F256). **e** Acetylcholine molecules (spherical presentation in red) preferably occupy the ligand-binding sites at the α (light blue)-γ(green). **f** In agreement with experimental studies, the second binding pocket of acetylcholine is located at the α (light blue)-δ (purple) interface area. In particular, the MD simulation shows higher density distribution in a close proximity to the aromatic residues, αTyr 93, αTrp 149, αTyr 190, αTyr 198, and εTrp 55/δTrp 57 that have been reported to relate to acetylcholine binding

In total over 2 μs of all-atom molecular dynamics simulations of the nAChR were conducted to generate verifiable hypotheses on potential molecular targets for ethanol. In the presence of different ethanol concentrations (25, 50, and 200 mM), the average three-dimensional density distribution robustly reveals a focal enrichment of ethanol molecules within an extracellular pore-adjacent pocket located between residues α65 and α90 (Fig. 1a, c; Supplementary Fig. 1A). Low, moderate, and high ethanol concentrations induce comparable conformational fluctuations on nAChR within the 100 ns simulation time (Supplementary Fig. 1B). The density distribution maps are averages overall frames of the 100 ns simulation time and all ethanol molecules and as such, do not reflect the occupancy probabilities of individual ethanol molecules. However, tracing the trajectories of ethanol molecules suggests that the high focal density is not only due to ethanol molecules moving in and out the pocket but is also a consequence of ethanol molecules occupying the area for some time in close proximity to α65 (Torpedo numbering) for a period of time (Supplementary Fig. 1C). In addition, molecular docking analyses suggest that ethanol may form a hydrogen bond (H-bond) of −2.0 kcal/mol with the (neutral) backbone carbonyl oxygen of the pore-residing (negatively charged) aspartic acid α83 (Fig. 1b; Supplementary Fig. 2). Ethanol molecules can attract other ethanol molecule and water through H-bonds. The non-polar ends of ethanol would attract non-polar molecules such as those found in gasoline through induced dipole interactions (London forces). Here, there is no direct interaction with the acidic side chain of aspartic acid however, its negatively charged carboxylate is necessary to induce a dipole by organizing the electron density required for building a hydrogen bond between the backbone oxygen and ethanol. Sequence alignment shows a high degree of homology of the D*VW motif among members of the Cys-loop receptor superfamily, which are known to interact with ethanol at different concentrations^9–16^, suggesting a negative charge at positions equivalent to α83 may be a shared site for ethanol interaction. In silico mutagenesis of α83 to lysine (K), followed by molecular dynamics simulations, show a diminished attraction of ethanol molecules toward the D*VW motif (Fig. 1a), and analyses of the simulation via heat maps reveal a marked reduction in ethanol-induced conformational changes (Fig. 1c). In addition, the radial distribution function *g(r)* for ethanol molecules (Supplementary Fig. 3) shows a lack of selectivity toward the mutated αD83K receptor in distances r larger than the interaction cutoff of the simulations, namely *r* > 9 Å.

**Fig. 2:**
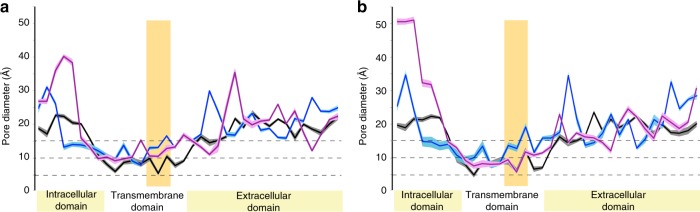
Fluctuations in the pore diameter of the nAChR during simulation. **a** Fluctuations in the pore profile of wild-type nAChR in presence of water (black), ethanol (pink), and acetylcholine (blue) are presented as averages over final 10 ns (shades indicate SEM). Pore diameter in the ligand free remains robust, whereas 200 μM ACh induces considerable fluctuations and increases along the channel. A total of 25 mM of ethanol induces diverse fluctuations in extracellular and transmembrane domain. **b** The pore of the αD83K mutant remains unchanged in the presence of ethanol. In the ligand-free, as well as ACh simulations pore profiles are comparable with WT. However, ethanol-induced fluctuations in the extracellular and transmembrane domains are marginal in comparison to WT receptor

Simulations of the wild-type nAChR in the presence of ACh reveal a gradual increase in the diameter of the hydrophobic barrier (Fig. 2a, b), accompanied by an approximately twofold increase in the pore volume (Table 1). Thereby, ACh preferably occupies the ligand-binding sites at the interfaces of α-γ and α-δ/ε interface areas (Supplementary Fig. 4). In particular, the MD simulation shows higher density distribution in a close proximity to the aromatic residues, αTyr 93, αTrp 149, αTyr 190, αTyr 198, and εTrp 55/δTrp 57 (Fig. 1d) that have been reported to relate to acetylcholine binding^21–23^. The inner pore-facing helices rotate by 15° normal to the membrane surface, and these rotational movements break the hydrophobic girdle, which widens the pore by almost 3 angstroms^24,25^. By contrast, the pore remains essentially unchanged in the ligand-free setup. The gradient in the total energy of the system decreases from approximately −1.28 to −1.38 × 10^6^ kcal/mol. At constant pressure, this change equals the energy transfer of the receptor with the environment through heating or work, other than expansion work. Thereby, it mostly reflects variations in the potential energy of the nAChR from the relaxed and stable closed state into the fluctuating state that may require stronger intermolecular forces to maintain its stability. Thus the simulations recapitulate the experimentally observed process in which binding of ACh induces a local perturbation in the ligand-binding domain that is linked to the 35 Å distant membrane domain by an axial rotation of both α-subunits^24,25^. Simulation results of wild-type AChR in presence of both ethanol and acetylcholine did not differ from the findings associated with ACh alone (Supplementary Fig. 5). In particular, the presence of ACh did not influence the high-density distribution of ethanol in the proximity of α83 (Supplementary Fig. 1A). These observations are indicative of conformational changes toward a state transition by ACh. However, the simulation time of 100 ns is not sufficient to reveal a complete receptor opening as well as experimentally observable ethanol-induced changes in nAChR conformation associated with ion flux and receptor function.

**Table 1:** Computational estimations of changes in pore volume of WT and mutant neuromuscular nAChR

Receptor/ligand	Receptor pore volume (Å^3^)	Volume % Apo-form	Volume % average ethanol-induced changes
**nAChR/H**_**2**_ **O**	54,693	100%	
**nAChR/ACh**	99,262	181%	
**nAChR/EtOH (25** **mM)**	73,506	134%	100%
**nAChR/EtOH (50** **mM)**	72,180	132%	100%
**nAChR/EtOH (200** **mM)**	72,110	132%	100%
**nAChR/EtOH (averaged)**	72,599	133%	100%
**nAChR/ACh** **+** **EtOH**	100,018	183%	136%
**D83K/H**_**2**_ **O**	56,982	104%	
**D83K/EtOH**	50,428*	92%	69%
**D83K/ACh**	98,551	180%	
**D83K/ACh** **+** **EtOH**	99,912	182%	198%
**D83S/EtOH**	50,773*	93%	69%
**D83E/EtOH**	68,019	124%	93%

Substitutions of aspartic acid (D) at α83 with lysine (K), serine (S), and glutamate (E), in presence of only ionized water (H2O), acetylcholine (ACh), and different concentrations of ethanol (EtOH) show a sensitivity of ethanol interactions to negative charge. Asterisks (*) denote significant differences in ethanol-induced changes of pore volume in mutants in comparison to WT receptor

Simulation of the wild-type AChR in the presence of ethanol alone shows small conformational fluctuations and an average increase of the hydrophobic barrier diameter to ~4 Å, accompanied with decreases in pore diameter in extracellular domain (Fig. 2a). For all ethanol concentrations, the pore volume increases to 133% of the closed state (Table 1), that is mostly associated with fluctuations within the intracellular domain. However, the holo-state pore shows fluctuations ranging from moderate expansion to large contractions (Supplementary Fig. 6). Therefore, the increase in pore volume within the 100 ns simulation time does not necessarily reflect a higher receptor permeability and ion flow through the pore. Nonetheless, in agreement with the density distribution maps showing a lack of attraction of ethanol to the αD83K mutant, ethanol also does not induce any remarkable fluctuations in the pore diameter (Fig. 2c) and volume (Table 1).

Simulations of the charge-conserved αD83E mutant (equivalent to rat and human α83) nAChR in the presence of ethanol reveal similar changes in pore volume compared to the wild-type nAChR, whereas analyses of the αD83S mutant nAChR and thus, neutral charge at α83 reveal no ethanol-induced changes (Table 1), and the distribution of ethanol molecules throughout simulation space remains uniform. Therefore, a negative charge at position α83 appears required for interactions of ethanol molecules with nAChRs. Furthermore, the simulations show that ethanol insensitivity is not induced by changes in the solvent accessible surface areas (Supplementary Table 1).

Thus the overall findings suggest that ethanol interacts with the negatively charged amino acid at α83, whereas ethanol is without effect on the αD83K mutant (Supplementary Fig. 7).

### Single channel recordings

**Fig. 3:**
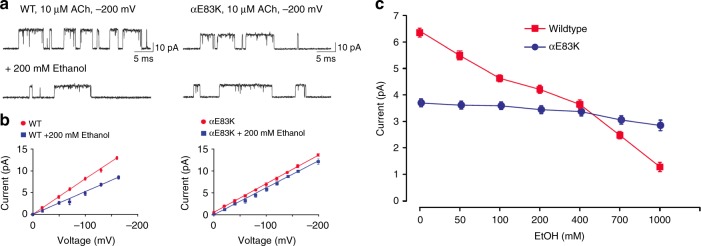
Ethanol reduces the single channel current amplitude in wild type but not mutant nAChRs. **a** Single channel currents elicited by 10 μM ACh, in the absence (upper traces) or presence (lower traces) of 200 mM ethanol at a membrane potential of −200 mV, are shown at a bandwidth of 10 kHz. **b** The mean current amplitude is plotted against membrane potential in the absence (red) or presence (blue) of 200 mM ethanol for the wild type (left) or mutant (right) nAChRs. Mean current amplitude was determined by analysis of all-points histograms (Methods) from 3 to 5 patches for each experimental condition. Unitary conductance determined (pS) from the slope of the current–voltage plots: wild type, 74.3 ± 1.4; wild type + 200 mM ethanol, 45.4 ± 1.8; αE83K, 59.5 ± 0.7; αE83K + 200 mM ethanol, 54.2 ± 0.2. **c** Shows the effect of different, physiologically relevant ethanol concentrations on the single channel current amplitude for wild type (red) or mutant (blue) nAChRs. Single channel currents elicited by 10 μM ACh were recorded at a membrane potential of −0 mV. Mean current amplitude was determined by fitting a Gaussian function to histograms of detected single channel openings from 3 to 5 patches for each experimental condition. Error bars indicate the SD

To evaluate the in silico predictions in vitro, we recorded ACh-elicited single channel currents from the adult human nAChR without and with ethanol. In the presence of 200 mM ethanol, the unitary current amplitude for the wild-type AChR is markedly reduced (Fig. 3a); after removal of ethanol and washing with control buffer the unitary currents approach the control amplitude (Supplementary Fig. 8A). When examined over a range of membrane potentials, the reduction in amplitude seems to arise through a decrease in the slope conductance without change in the reversal potential (Fig. 3b). By contrast, the unitary current for the αE83K mutant AChR (equivalent to Torpedo αD83K used for simulation) in the presence of ethanol is only slightly reduced (Fig. 3a). Recordings from the αE83K mutant AChR, over a range of membrane potentials, reveal that neither the slope conductance nor the reversal potential change in the presence of ethanol (Fig. 3b). Thus ethanol, at a concentration of 200 mM, markedly reduces the unitary current through the wild type but not the mutant AChR channel.

We next evaluated whether ethanol reduces the unitary current over a physiologically attainable concentration range. For the wild-type human nAChR, at a membrane potential of −70 mV, increasing the ethanol concentration produces a biphasic reduction of the unitary current (Fig. 3c). The first phase shows a linear decrease in current between 0 and 100 mM ethanol, and corresponds to a physiologically attainable ethanol concentration. The second phase between 400 and 1000 mM is not considered physiologically attainable. By contrast, nAChRs bearing the αE83K mutation show little reduction in the unitary current over the same range of ethanol concentrations as predicted by simulations. Interestingly, the second phase, which might be considered a non-specific effect of ethanol on the membrane, is not observed in the mutant. Thus, physiologically attainable concentrations of ethanol reduce the unitary current through the wild type but not the mutant nAChR.

By contrast, a concentration of 200 mM ethanol did not affect ACh-elicited channel open and closed dwell times. An overlay of the open time histograms from recordings in the presence and absence of ethanol shows that the two distributions coincide closely (Supplementary Fig. 8B). In particular, the major exponential component representing receptors with two bound agonists shows no significant change (*p* = 0.443). Thus, ethanol does not appreciably alter the energetic barrier between doubly occupied open and closed states. Similarly, an overlay of the closed time histograms from recordings in the presence and absence of ethanol shows that the two distributions coincide closely (Supplementary Fig. 8B). Thus ethanol does not appreciably alter energetic barriers associated with agonist mediated conformational changes that precede channel opening.

Together, the in silico and in vitro studies suggest that ethanol interacts with an extracellular region of the nAChR in the vicinity of αE83 (and αD83). Moreover, the in vitro investigation suggests that this interaction reduces the rate of ion flow through the open channel. Previous studies^26,27^ showed that multiple rings of polar or negatively charged residues line the ion translocation pathway and thereby contribute to charge selectivity and amplitude of the ionic current. The reduction of the unitary current by ethanol may thus arise through its interaction with the extracellular charged ring that encompasses αE83.

### Ethanol effects on isolated muscle

**Fig. 4:**
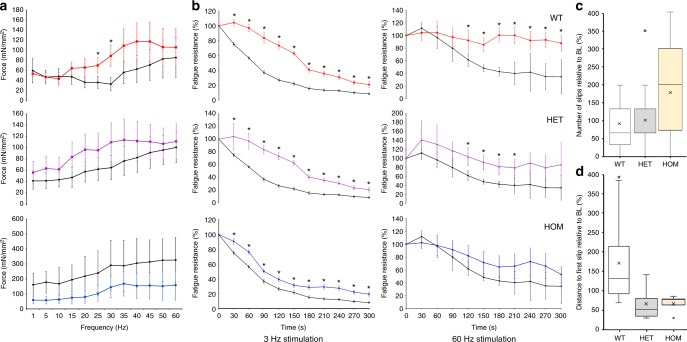
Force-frequency relationship and fatigue resistance of EDL muscles by electrical stimulation of the peroneal nerve, and tests of motor coordination in vivo. **a** Force-frequency protocol in EDL muscles from stimulated peroneal nerve (six muscle from *n* = 6/genotype). Ethanol increases force production in WT animals (red) in a frequency-dependent manner, in contrast to control conditions (black, top), particularly, at stimulation frequencies of 25 and 30 Hz (*p* < 0.05). Qualitatively EDL muscle of HET shows a similar force-frequency relationship, whereby ethanol increases (not significant) the force (purple) in contrast to the muscle under control conditions (black, middle). In contrast, ethanol decreases force in EDL muscle of HOM animals (blue) in comparison with control conditions (black, bottom). **b** The black time-series in each panel represents the decreasing course of fatigue resistance under control conditions normalized to the first measurement point. At low stimulation frequency of 3 Hz, ethanol enhances resistance to fatigue in muscle from all three genotypes WT (red), HET (purple), and HOM (blue) animals significantly (*p* < 0.05). In contrast, at high frequencies muscle from HOM mutant animals (blue) does not respond to ethanol, leading to a decreasing fatigue resistance that does not differ significantly from control conditions (black). EDL muscles contained predominantly fast type 2B fibers. Thus, the 60 Hz stimulation is the functionally relevant frequency. In the presence of ethanol, WT and HET did not differ significantly. In contrast, in the HOM genotype (blue) the effect of ethanol in 60 Hz protocol was not significantly different from the controls (*p* = 0.32). **c** Motor impairment measured as the number of slips in the ladder rung walking test 10 min after acute administration of ethanol in WT (*n* = 13), HET (*n* = 14), and HOM (*n* = 15) CHRNA1 mutant rats. The data are normalized to baseline (BL) measurements and demonstrates a twofold less slip, yet not significant (*p* = 0.12), in WT and HET animals following ethanol administration. **d** Ethanol prolonged the distance to first slip in WT animals significantly in comparison to HET (*p* < 0.05) and HOM (*p* < 0.05) animals. In average, ethanol injection lead to a 2.2 times longer distance in WT in comparison to HOM rats

To test whether mutating αE83 affects skeletal muscle function in the presence of ethanol, we introduced the E > K mutation into the rat genome using the CRISPR/Cas9 system (Supplementary Fig. 9). We isolated the extensor digitorum longus (EDL) muscle together with the peroneal nerve of CHRNA1 transgenic rats, and measured force production upon electrical stimulation of the nerve. The peroneal nerve was stimulated for 500 ms at frequencies of 1, 5, 10, 15, 20, 25, 30, 35, 40, 45, 50, and 60 Hz with a 3 min break between stimulations. Each muscle was treated either with control solution or with 25 mM ethanol. Initially, we measured force production over this range of stimulation frequencies (Fig. 4a).

In control physiological saline, force production increases with increasing stimulation frequency until reaching a plateau of 60 Hz, as reported previously^28^. In the presence of 25 mM ethanol, force production increases beyond that in control saline for wild type (WT) and heterozygous (HET) animals. However, it decreases compared to control saline in homozygous (HOM) mutant animals, suggesting the mutation prevents the enhancement of force production by ethanol. Thereby, a potentially perplexing feature of these measurements is the seemingly higher force production levels in HOM muscle under control conditions in comparison to WT and HET. In addition, the variability in tetanic force generation appears greater in HOM than either WT or HET. However, statistical comparison of the genotypes shows no significant differences at any stimulation frequency (*p*_1 Hz_ = 0.27; *p*_5 Hz_ = 0.29; *p*_10 Hz_ = 0.32; *p*_15 Hz_ = 0.28; *p*_20 Hz_ = 0.30; *p*_25 Hz_ = 0.27; *p*_30 Hz_ = 0.25; *p*_35 Hz_ = 0.29; *p*_40 Hz_ = 0.30; *p*_45 Hz_ = 0.33; *p*_50Hz_ = 0.36; *p*_60 Hz_ = 0.37). Moreover, the opposing effect of ethanol between WT and HOM animals, although seemingly clear, was not significantly different owing to the large range of force production at each frequency. Therefore, we implemented a second protocol in which fatigue resistance of EDL muscle was measured as a function of time (Fig. 4b). The influence of ethanol on the resistance to fatigue response was evaluated as the effect size (ratio of fatigue resistance for ethanol and control solution) for each genotype and then normalized to the first response.

In control saline, force production declines exponentially with time at both stimulation frequencies for WT, HET, and HOM animals. At low stimulation frequency of 3 Hz and in the presence of ethanol, force production declines more slowly and to a reduced extent in all three genotypes significantly (*p* < 0.05). However, since EDL muscles largely contain fast type 2B (75.7 ± 2.2%)^28^ fibers, the behavior of the muscle at low frequencies might provide functionally relevant information. Thus, the fatigue evolution in the EDL muscle in presence of ethanol was further investigated at high frequency stimulation of 60 Hz. Hereby, ethanol significantly reduced fatigue development in WT and HET animals (*p* < 0.01); whereas, the decline in force production is not significantly altered in HOM animals (*p* = 0.32). In addition, regression analysis of the time-series suggests that ethanol induces a significant reduction of fatigue in WT, in both the level and duration, in 3 Hz and 60 Hz protocols (*p* < 0.05 and *p* < 0.001). Thus whereas ethanol reduces muscle fatigue in WT and HET animals, it is without effect in HOM animals.

### In vivo assessment of ethanol effects on muscle function

The three genotypes exhibited no differences in home-cage locomotor activity (*p* = 0.88), latency to cross the ladder (*p* = 0.61), diurnal, nocturnal, or circadian activity, nor did they differ in tests of motor coordination, balance, or grip strength (Supplementary Fig. 10). Blood alcohol concentration was not significantly different between male and female WT, HET, and HOM animals measured after 30 (*p*_male_ = 0.34; *p*_female_ = 0.66), 60 (*p*_male_ = 0.27; *p*_female_ = 0.47) and 120 min (*p*_male_ = 0.65; *p*_female_ = 0.53). Following administration of ethanol (Supplementary Fig. 11), all three genotypes performed similarly in tests of locomotor activity (*p* = 0.14), latency to cross the ladder (*p*_5min_ = 0.39; *p*_10min_ = 0.12), equilibrium (*p* = 0.16), grip strength (*p* = 0.17), and inclined plate (*p* = 0.57).

However, the ladder-walking test, another measure of motor coordination, revealed a noticeable effect following administration of ethanol. Ethanol doubled the number of slips in HOM animals (Fig. 4c). While, due to animal-to-animal variability, the difference in number of slips between WT and HET or HOM animals fell short of statistical significance (*p* = 0.12), the relative risk (RR) of slips in HOM animals doubled. Moreover, the RR of the distance to the first slip was 1.89 (95% confidence interval [CI] 1.67–2.08) higher in HOM than WT animals, in accord with expectations from the muscle fatigue measurements. Moreover, ethanol increased the distance to the first slip in WT animals significantly (*p* < 0.05) in comparison to HET and HOM animals by 215.5% (Fig. 4d), congruent to the expectations from the muscle fatigue measurements.

## Discussion

The collective findings may be synthesized as follows. Atomistic computational studies reveal a novel site of ethanol action within the extracellular region of the nAChR that initiates voluntary muscle movement. This finding substantially challenges the current view on ethanol interactions with pentameric cys-loop receptors. While few recent studies have proposed alcohol binding sites located in the extracellular domain of GABA_A_ receptors^29,30^, along with an indirect site in the intracellular domain of glycine receptors^31^, the majority of investigations suggest that ethanol affects the function of pentameric cys-loop and particularly nACh receptors by influencing residues within the transmembrane domain^3–9^.

Our study provides converging evidence in favor of an extracellular site of action of ethanol with nAChR. Neuromuscular nicotinic acetylcholine receptors of different species (Torpedo^32^ and rats^33,34^) have been shown to interact with short chain n-alcohols (alkanols). n-alcohols such as propanol or decanol inhibit channel activity by interaction at just one hydrophobic site within the ion channel lumen. Yet, these studies failed to observe an inhibitory response of peripheral nAChR to ethanol at physiological concentrations and could only demonstrate such effect at concentrations above 300 mM^34^. Our study challenges these findings and is supported by previous reports of ethanol-induced inhibition of other members of Cys-loop ligand-gated ion channel family, such as ELIC^35^, ρ_1_-GABA_A_ receptors^36^, and nematode glutamate-gated chloride channels^37^. In particular, a systematic investigation of the dose-response relationship to n-alcohols^35^ shows that ethanol, while weaker than other alcohols, inhibits the pentameric ligand-gated ion channel ELIC even at lower concentrations.

Due to the high level of homology between the muscle nAChR and other members of cys-loop family that are expressed within the central nervous system, our findings may open new perspectives in understanding mechanisms of action of ethanol in the brain as well.

Patch clamp recordings of nAChR single channel currents verified the computational predictions by demonstrating that the substitution of α83 with a non-negative amino acid, such as lysine, would diminish the ethanol-induced effects, providing direct evidence for interaction of ethanol with the D*VW motif. Furthermore, the single channel measurements reveal that physiologically attainable concentrations of ethanol reduce the unitary current amplitude in wild type but not mutant nAChRs, and suggest a change in the nAChR’s global conformation.

Measurements of skeletal muscle force production show that physiologically attainable concentrations of ethanol enhance resistance to fatigue of EDL muscle in WT and HET animals, but are without effect in HOM animals. In other words, ethanol at low to moderate concentrations improves motor function in healthy muscles. A plausible mechanism combines the membrane hypothesis of muscle fatigue with the previously demonstrated interaction between the nAChR and the Na^+^–K^+^ ATPase. The membrane hypothesis of fatigue posits that repetitive stimulation increases the concentration of extracellular K^+^, which depolarizes the muscle sarcolemma and transverse tubular system^38^, and ultimately inactivates the action potential. The activity of the α2 isoform of the Na^+^–K^+^ ATPase has been shown to increase through physical interaction with conformations of the nAChR formed upon desensitization or in the presence of non-competitive blockers^39^, which reduces the concentration of extracellular K^+^ and hyperpolarizes the muscle fiber. The α2 isoform of the Na^+^–K^+^ ATPase represents the predominant α-subunit content in fast-twitch muscles and is known to maintain contraction and oppose fatigue in the working muscles^40^. We speculate that ethanol promotes an analogous conformation of the nAChR, which in turn increases its interaction with the α2 Na^+^–K^+^ ATPase, increasing its activity, which reduces extracellular K^+^, hyperpolarizes the muscle fiber^41^ and reduces muscle fatigue.

These findings are fortified by our analysis of motor activity and coordination of WT and mutant animals. Muscle fatigue is associated with a reduced motor coordination, thus slipping in the ladder walk test relates in vivo function directly to the ex vivo measurements. In accord with the ex vivo findings, this test further shows ethanol-induced improvements in motor function, as ethanol at low to moderate concentrations, reduced not only the total number of slips but also the risk of slippage in WT but not HOM animals. These observations provide evidence at the living organism level that ethanol effects on the muscle function are not solely mediated by the central nervous system, but also by an interaction with a specific site in the water accessible extracellular domain of the neuromuscular acetylcholine receptor. Thus combining atomistic computational, single molecule electrophysiological, transgenic animal model, and physiological approaches converges upon a novel molecular mechanism behind one of ethanol’s multiple physiological consequences.

Although, the multi-disciplinary approaches have converged into a viable mechanistic framework, there are some unavoidable limitations. Molecular dynamics simulations are generally powerful tools to improve our understanding of atomistic processes, interactions and fluctuations in biomolecular systems that at their thermodynamic limits can be measured experimentally. We used the molecular dynamics simulations as a tool for generating hypotheses on the site of action of ethanol at nAChR. However, there is a strong stochastic element in the simulations. While an increase in the number of ethanol molecules or simulation time would certainly enhance the probability to identify the site of action of ethanol at the receptor, the current discovery might have been rather due to fortuitous initial conditions. Moreover, there is usually a mismatch in spatiotemporal scales of consideration between simulation and experiment. Molecular dynamics simulations are often restricted to a single macromolecule, simulated for tens or hundreds of nanoseconds, whereas experiments investigate numerous proteins for periods of microseconds to minutes. Thus, complete simulations of conformational transitions, as in this case, are often not possible.

In addition, the structural models of the simulated proteins are often not available or incomplete, which may lead to discrepancies between the in silico predictions and experimental validations. At the time this study was initiated, the refined model of the membrane-associated Torpedo (α_1_γα_1_β_1_δ) acetylcholine receptor (nAChR receptor) at 4 Å resolution^20^ was the accepted model. Our main rationale for using the *Torpedo* nAChR for molecular dynamics simulations was that it is the only pentameric ligand-gated ion channel (pLGICs) structure to have been determined in a lipid membrane environment. This may be important in preserving the native structure of the transmembrane domain.

Our approach does not correct for an error in the assignment of amino acids *Torpedo* nAChR density map within the second and third transmembrane (TM) helices (TM2 and TM3)^42^. Such a correction was made by a recent study^43^ and would advance the protein chain by four residues from M2 to the end of the protein. Therefore, it must be noted that there are differences between the Torpedo model used for simulation and the true structure of the muscle AChR. However, analysis of Ramachandran plots shows that in our simulations, the structural integrity of the receptor is largely provided. We have visualized energetically allowed regions for backbone dihedral angles ψ against φ of amino acid residues in *Torpedo* protein structure (Supplementary Fig. 12). The Ramachandran plot of 2BG9 model^20^ shows that number of residues found in the favored region 81.7% from the total residues, and number of residues in allowed region is 12.9% and number of residues in outlier region 5.4%. The starting conformation for MD simulations following energy minimization however shows clear improvements. The number of residues found in the favored region is 95.6% from the total residues, and number of residues in allowed region is 3.5% and number of residues in outlier region 0.09%. In spite of these improvements, even in the current structures, most of the cytoplasmic loop between M3 and M4 is still missing since its presence seems to prevent stable structures suitable for either X-ray diffraction or cryo-electron microscopy. Thus, the results of the molecular dynamics simulations shall be treated with caution and be considered not as hard facts but indications.

## Methods

### System setup for molecular dynamics simulations

Simulations were based on the refined model of the membrane-associated Torpedo (α_1_γα_1_β_1_δ) acetylcholine receptor (nAChR receptor) at 4 Å resolution (Protein Data Bank accession number 2BG9^20^). This atomistic model represents the whole receptor in a closed-channel conformation. The nACh receptor was inserted into an equilibrated, fully solvated lipid bilayer containing palmitoyloleoylphosphatidylcholine (POPC) membrane; lipids within 0.8 Å of the receptor protein were removed. In each simulation, the simulation box initially measured 200 Å × 200 Å × 180 Å and contained one nACh receptor, ~530 lipids, ~65 sodium ions, and ~174,874 water molecules, for a total of ~696,630 atoms (Supplementary Fig. 13). Two control simulations (each 100 ns) were designed to investigate the robustness of the membrane for the present study: (1) the water-solvated, charge-neutralized 200 Å × 200 Å POPC membrane was simulated to ensure membrane stability; (2) the water-solvated, charge-neutralized 200 Å × 200 Å POPC membrane was simulated in presence of 25 mM ethanol molecules to ensure membrane stability and absence of ethanol-induced phase transitions of the membrane. There were no noticeable changes in the membrane structure.

### Simulation protocol and force field parameters

All molecular dynamics simulations were performed using the highly scalable parallel simulation code NAMD 2.9^44^ and the modified CHARMM27 parameter set for proteins, lipids, ethanol, and salt ions and the CHARMM TIP3P model for water^45^. For energy minimization, the conjugate gradient method for 15,000 time-steps (15 ps) was applied. Here, a 200 ps NpT equilibration was conducted, which is sufficient for the temperature of the system to reach a stable plateau of 310 °K (Supplementary Fig. 14). The choice of this temperature was based on the phase transition temperature of the POPC lipids. For all simulations the non-bonded interactions had a short cutoff of 9 Å, and periodic boundary conditions with Particle Mesh Ewald method to account for long range electrostatic interactions^46^ were used. Langevin dynamics and a Langevin piston algorithm were used to control the temperature and pressure of the system and maintain the ensemble at 310 K and a pressure of 1 atm. All production simulations were initiated from the final snapshot of each corresponding equilibration run, and were performed for 100 ns. In total, the simulations took over 3 million processor hours.

### Simulation of ligand-receptor interactions

Six different conditions were simulated using the setup for the complex of membrane and refined wild-type nAChR. The membrane-receptor complex was simulated to ensure conformational stability of the ligand-free receptor in the closed state. Physiological concentrations of acetylcholine (0.2 mM^47^) were applied to the system to simulate the agonist-induced conformational changes of the receptor channel. By matching the averaged density distribution maps of ACh with the experimentally determined ACh binding sites at the α-γ and α-δ subunit interfaces, this simulation serves as a proof of concept for the setup to investigate ligand–receptor interactions (Fig. 1 and Supplementary Fig. 4). Ethanol concentrations of (25, 50, 200) mM were applied to the solution to simulate the pure ethanol-induced effects on the native receptor. Ethanol and acetylcholine were added at concentrations of 25 mM and 0.2 mM to investigate their interactions together and in combination with the receptor. Ethanol and acetylcholine molecules were inserted into the mixtures using PACKMOL^48^ by random distribution within the extracellular space within at least 20 Å of the membrane-protein complex.

### Design and simulation of mutated receptors

We performed simulations of ethanol interactions with several nAChR mutants we designed—on the basis of wild-type receptor simulations and docking analysis (see below)—to probe the key role of αD83 on the alteration of the pore diameter and volume. These simulations (Table 1), were performed as described above. The mutated residues were modeled using the PyMOL mutagenesis wizard^49^. Simulations containing the mutations D83S, D83E, and D83K were designed to explore how the electric charge of the amino acids affect the ethanol-induced conformational changes, and at the same time whether a point mutation is sufficient to diminish the ethanol effects. To further address the latter question and investigate interspecies universality of the observations, simulations containing the mutations D83Q and D83G as well as three cavity substitutions (DDVW cavity with human α_1_ EKIW and human α_4_ ELIW as well as with human α_7_ GQIW) were performed (results not reported). In addition, 0.2 mM of acetylcholine was applied to the D83K mutated receptor to demonstrate that the mutation does not affect the ability of the receptor channel to open.

### Analysis protocols

Trajectory frames (atomic coordinates) were saved every 100 ps during production simulations. VMD^50^ was used to visualize trajectories and render molecular images. The root mean squared deviation (rmsd) of the protein backbone, as well as rmsd of all protein residues was calculated and represented as heat maps. Spatial density distributions of ligands were calculated using the Volmap plug-in of VMD with a resolution of 1 Å, and averaged over the entire simulation period (100 ns). Average diffusion coefficients of ethanol and ACh molecules were calculated using the Einstein relation. The radial distribution function, *g*(*r*) = *ρ*4*πr*^2^*dr*, describes the variations of density of ethanol molecules *ρ* as a function of distance *r* from a reference amino acid in the receptor. Thus, it can be presented as a probability histogram of finding an ethanol particle at a distance of *r* from amino acids in position α83 of different WT and mutated nAChR. While *g*(*r*) is in general calculated for *r* > 0, its interpretation is limited to distances above the cutoff of the non-bond interactions in the simulations, which in present study was set as 9 Å. Within this range, it provides a robust measure to describe the efficacy (i.e., lack of interaction with ethanol molecules) of point mutations with non-negative amino acids. nAChRs are cation-selective ion channels, thus the fluctuations in pore size may correspond to interactions with ligands. Porewalker^51^ and 3Vee^52^ were used to calculate pore size (i.e. diameter and accessible volume) for all WT and mutated receptors for the interval [90, 100] ns of simulations and averaged. Pore radii of the trajectories were computed using HOLE software^53^, extracted each nanosecond. The NAMDenergy plug-in of VMD, as well as a control averaging of the total energies of the backbone structure of the receptor proteins, were used to estimate the average energy levels of WT and mutated receptors (in kcal/mol). All calculated values were statistically compared with respect to the time-steps by one-way analysis of variance (ANOVA) with the Holm-Bonferroni correction method with a global level of significance of *α* < 0.05 to identify significant heterogeneity factors using IBM SPSS statistics 21. ClustalX^54^ was used for sequence alignment and homology analysis (Supplementary Fig. 15). In order to evaluate the structural integrity of the *Torpedo* model, Ramachandran diagrams were generated and analyzed by RAMPAGE server (http://mordred.bioc.cam.ac.uk/~rapper/rampage.php). As discussed in previous studies^55^, these plots represent *phi* versus *psi* dihedral angles for every residue in the protein. The diagram is divided into favored, allowed, and disallowed regions, whose contouring is based on density-dependent smoothing for 81,234 non-Glycine, non-Proline residues with *B* < 30 from 500 high resolution protein structures. Regions are also defined for Glycine, Proline, and pre-Proline.

### Docking protocol

MD simulations of the interaction between ethanol and the nAChR suggest the αDDVW cavity is a target for ethanol action. Due to thermodynamic fluctuations during the simulation, it is hard to robustly predict the “binding” sites of alcohol in terms of single amino acids solely based on the MD-based density distributions of ethanol molecules. Thus, AutoDock Vina 1.1.2^56^ was used to further specify potential amino acid targets of ethanol within the αDDVW cavity. Hydrogen atoms were added to the nAChR protein (PDB: 2BG9), and the structure file was converted to PDBQT format using AutoDockTools 1.5.6^57^. The search space volume was chosen as 8000, 27,000, and 64,000 Å^3^ such that at maximum size it largely encompassed the entire receptor (receptor volume was approximated as a cylinder: 50,240 Å^3^). The latter serves as a control docking experiment and probes the possibility of cavities with higher affinity than αDDVW. The exhaustiveness value was set as 1000. Docking scores were calculated and ranked with respect to different search spaces. The results achieved with 8000 or 27,000 Å^3^ box did not differ qualitatively from the results of control docking experiments using a 64,000 Å^3^ box. The highest ranked docked pose of ethanol (congruent to molecular dynamics results) was obtained, and the involved amino acid αD83 was used as the target of in silico and in vitro mutagenesis.

### Expression of adult human AChRs

Complementary DNAs encoding adult human α, β, ε, and δ-subunits, installed within the cytomegalovirus expression vector pRBG4, were transiently transfected into the BOSC 23 clonal cell line. The mutation αE83K was generated using the QuikChange site-directed mutagenesis kit (Agilent Technologies), and the presence of the mutation and absence of unwanted mutations were confirmed by sequencing the coding region. Cells were maintained in Dulbecco’s modified Eagle’s medium containing 10% (v/v) fetal bovine serum at 37 °C until they reached 50% confluence. Thereafter, cDNAs encoding either wild type or mutant AChR subunits, plus a cDNA encoding green fluorescent protein, were transfected by calcium-phosphate precipitation. Patch clamp recordings were made 12–48 h following transfection^58^.

### Single channel recordings

Recordings were obtained in the cell-attached patch configuration^59^ at a temperature of 21 °C, essentially as described previously^58^. Patch pipettes were pulled from glass capillary tubes (No.7052, King Precision Glass) and coated with Sylgard (Dow Corning). The bath solution contained (in mM): 142 KCl, 5.4 NaCl, 1.8 CaCl_2_, 1.7 MgCl_2_, and 10 HEPES, with the pH adjusted to 7.4 by addition of KOH. The pipette solution contained (in mM): 142 NaCl, 5.4 KCl, 1.8 CaCl_2_, 1.7 MgCl_2_, and 10 HEPES, with the pH adjusted to 7.4 by addition of NaOH. Specified concentrations of acetylcholine chloride, with or without ethanol, were added to the pipette solution. Before establishing a cell-attached patch, the pipette offset potential was manually zeroed, and following formation of a giga-ohm seal, a defined membrane potential was established via a command voltage applied to the interior of the patch pipette. Single channel currents were recorded using an Axopatch 200B patch clamp amplifier (Molecular Devices) with the gain set at 100 mV/pA and the internal Bessel filter at 100 kHz. The current output was sampled at intervals of 2 μs using a National Instruments model BNC-2090 A/D converter with a PCI 6111e acquisition card, and recorded to the hard disk of a PC computer using the program Acquire (Bruxton).

### Single channel analysis

Single channel analysis was performed using the program TAC 4.2.0 (Bruxton), which digitally filters the raw data (Gaussian response), and interpolates the digitized points using a cubic spline function^60^. Two methods were used to determine the single channel current amplitude, both of which used procedures within TAC. In the first method, with the Gaussian digital filter set at 5 kHz, an all-points histogram was generated for each sweep of data from a given patch, and two Gaussian functions, one for the baseline and the other for the open channel current, were fitted to the histogram. The difference between the baseline and open channel currents defined the mean current amplitude of each sweep, and the mean current for at least twenty sweeps was averaged to yield the overall mean current amplitude. In the second method, single channel openings from an entire recording were detected using the program TAC 4.2.0 (Bruxton) with the Gaussian digital filter set at 5 kHz and the detection threshold set at half the unitary current amplitude. To determine the mean current amplitude, a histogram of current amplitudes from greater than 1000 detected channel openings was generated, and then fitted by a Gaussian function.

### Design of gRNAs and functional test

We used the online tool CRISPOR (http://crispor.org) for the selection of the *Chrna1* guide RNA (gRNA) target sites as well as for the off-targets prediction^61^. Three gRNA target sites with high-specificity scores were chosen, two of them directly binding at the site where we aimed to introduce the mutation and one further downstream. For identification of the most effective Cas9 target site, dual expression vectors were commercially obtained, coding for the selected *Chrna1* gRNAs and Cas9 (Genescript). The *Chrna1* gRNA target regions were inserted into a nuclease reporter plasmid (pTAL-Rep^62^) using annealed synthetic oligonucleotides in between a partly duplicated, non-functional β-galactosidase gene. HeLa cells were transfected with a combination of one of the dual expression vectors and the *Chrna1*-specific reporter plasmid. After transfection the Cas9-nuclease-induced double-strand breaks stimulated the repair of the *lacZ* gene segments into a functional reporter gene, the activity of which is determined in cell lysates using an o-nitrophenyl-β-D-galactopyranosid (ONPG) assay. A luciferase expression vector was also added to the transfection mix and luciferase activity was measured as transfection control^61^.

### Generation of genome edited rats

Wistar rats (Envigo) were bred in standard cages (Tecniplast) in a 12-h light/dark cycle in a temperature-controlled environment with free access to food and water in the animal facility of the Central Institute of Mental Health, Mannheim. All animal protocols were approved by the Regierungspräsidium Karlsruhe. Wistar one-cell embryos were injected using standard microinjections procedure. In brief, microinjections were performed in the cytoplasm and male pronuclei of zygotes with a mixture of Cas9 mRNA (15 ng/μl), sgRNA expression vector (6 ng/μL) and the oligonucleotide 5′-ATGGAATCCAGATGACTATGGAGGAGTGAAAAAAATTCACATCCCTTCGAAAAAGATCTGGCGGCCGGACGTTGTTCTCTATAACAAGTAAGCAAAACCATGC -3′ (10 ng/μl) as repair substrate. The injected embryos were cultured in M2 Medium at 37 °C in 5% CO_2_ and 95% humidified air until the time of injection. Surviving oocytes were transferred to the oviducts of pseudopregnant female rats.

### Transgene analysis

DSBs introduced in the genomic DNA by the CRISPR/Cas9 system are either repaired by imprecise Non-homologous end joining (NHEJ), leading to genomic microdeletions or insertions (indel mutations) or less frequently by homology directed repair (HDR) if a DNA repair template is available. By measuring the frequency of repair events in the target locus the efficiency of the CRISPR/Cas9 system can be analysed. NHEJ-mediated indel mutations can be detected by the T7 endonuclease I assay^63^. In brief, the *Chrna1* locus was amplified by PCR from 100 ng genomic tail DNA of animals born after microinjection with the primers Chrna1_check_for (*5*′ *CACTGATTCGGCCTGCTAAC 3*′) and Chrna1_check_rev (5′-GCCCTGATTCAATTTGGCTTCC-3′). The amplified 330 bp fragments were precipitated and ethanol dissolved in 25 µl buffer 2 (NEB). A total of 15 µl of the PCR product from wild type and CRISPR/Cas9 injection derived animals were combined and subsequently melted and reannealed using the following program: 95 °C for 10 min, 95–85 °C @ −2.0 °C/s, 85 °C for 60 s, 75 °C for 60 s, 65 °C for 60 s, 55 °C for 60 s, 45 °C for 60 s, 35 °C for 60 s and 25 °C for 60 s with a 0.3 °C/s decrease rate in between steps from 85 °C to 25 °C. This procedure partially leads to the alignment of mutant and wild-type DNA strands harboring mismatches (duplexes). Ten microliters of duplex DNA was digested with 1 µl T7 nuclease (NEB), which cleaves DNA duplexes at the sites of bulges or mismatches. The reaction was incubated at 37 °C for 15 min and then separated on a 10% TBE polyacrylamide gel. Ten microliters undigested duplex DNA was loaded as control. The gels were stained with ethidium bromide. In addition, *Chrna1* amplicons were also used for restriction enzyme digestion. The sequence of the oligonucleotide used for HDR was designed in such a way that *Chrna1* E > K mutation should be introduced together with a non-coding mutation to generate a new BstBI (TTCGAA) recognition site. PCR amplicons were incubated at 37 °C for 30 min with 1 µl BstBI (NEB) and then separated on a 10% TBE polyacrylamide gel. After successful integration of the oligonucleotide, the 330 bp PCR product was cleaved to give 113 and 217 bp fragments.

### Cloning and sequencing of the *Chrna1* target locus

Chrna1 locus fragments were obtained from founder tail DNA by PCR. The amplified fragments contained a pool of edited or non-edited target alleles from an individual animal. The PCR products were inserted into the pre-linearized vector pJet1.2 (Thermo Fisher). A total of 5–10 clones derived from each PCR amplicon were used to identify homologous recombination events at the targeted genomic Chrna1 site by DNA sequencing.

### Specificity of the genomic modification

The CRISPOR program was used to identify potential off-target sites for the selected *Chrna1 gRNA* 5′-CGGCCGCCAGATCTTTTCCG-3′. No off-target sites were detected in an exonic sequence on the same chromosome. The top 13 predicated off-target sites were located in intergenic or intron regions, with specificity scores ranking from 1.04 down to 0.19^64^, compared to the score of the target site of 94. From the CRISPOR off-target list, the two most important off-target sites in the founder DNA were chosen to be analyzed for the occurrence of indels by the T7 assay: the one with the highest off-target score in CRSPOR and the off-target site with the highest score was located in an exon (*Lrrc75b/Fam211b* gene). In addition, using the same PCR samples, we also applied the Surveyor mismatch-specific nuclease assay^65^, as this assay outperforms the T7 nuclease to detect single-nucleotide changes.

### Behavioral tests in *CHRNA1* mutant rats

Impact of CHRNA1 mutation on rat behavior was measured in basal locomotor activity tests, in the plus-maze anxiety test and in a series of motor coordination, balance and muscle-strength tests. All tests were performed in male and female wild type (*n* = 17), heterozygous (*n* = 19), and homozygous (*n* = 19) CHRNA1 mutant rats. Open field, motor coordination, balance and muscle-strength tests were repeated following the single intraperitoneal (i.p.) administration of ethanol. The time-line design of the experiments is presented in Supplementary Table 2.

### Animals

Fifty-six 2-month-old male and female Wistar rats were used for behavioral testing. All animals were housed in groups in standard rat cages (Ehret, Emmendingen, Germany) under a 12 h artificial light-dark cycle (lights on at 7:00 am). Room temperature was kept constant (23 ± 1 °C). Standard laboratory rat food (Ssniff, Soest, Germany) and tap water were provided ad libitum throughout the experimental period. Body weights were measured weekly. All animal experiments have been performed in accordance with the guidelines of the state of Baden-Wuerttemberg and have been approved by the Regierungspräsidium Karlsruhe.

### Home-cage activity

Home-cage activity was monitored by use of an infrared sensor connected to a recording and data storing system E-Motion (Infra-e-motion, Henstedt-Ulzburg, Germany)^66^. Rats were placed in single cages for 3 days and an E-Motion device was placed above each cage (30 cm from the bottom) so that the rat could be detected at any position inside the cage. The device sampled every second, whether the rat was moving or not. The sensor could detect body movement of the rat of at least 1.5 cm from one sample point to the successive one. The data measured by each E-Motion device was downloaded into a personal computer and processed with Microsoft Excel.

### Open field activity

To measure the locomotor activity in a novel environment, an open field apparatus was used^66^. An open field was a dark PVC box of four equal squares (51 × 51 × 50 cm). The light was adjusted to 50 lux (measured in the center of a square). All animals were habituated to the experimental room one day prior to the test. For the test, the animal was placed in the center of the box and the experimenter then left the room while a camera above the apparatus recorded the animal’s movements. Distance traveled was recorded digitally for a period of 60 min. The observation programme “Viewer” (Biobserve GmbH, Bonn, Germany) was used to analyse the behavior.

### Ladder rung walking test

The horizontal ladder rung walking test apparatus consisted of side walls made of clear plexiglas and metal rungs (3 mm diameter), which were inserted to create a floor with a minimum distance of 1 cm between rungs. The side walls were 1 m long and 19 cm high measured from the height of the rungs^67^. The ladder was elevated 50 cm above the ground with an enclosed safety box at one end and a start platform at the other end. The width of the alley was adjusted to the size of the animal, so that it was about 1 cm wider than an animal to prevent the animal from turning around. A regular pattern of the rungs (spaced at 1 cm intervals) was used for training sessions. An irregular pattern (distance varied from 1 to 5 cm) was used for testing. The latency to cross the ladder and the timing of limb protrusion between rungs when a miss occurred was recorded. Furthermore, the observation of limb protrusion was used to ensure that only full slips were taken into consideration. The entire procedure was additionally recorded by a video camera to analyse the distance and time associated with each slip.

### Equilibrium test

Equilibrium was assessed on a rough PVC beam (2.5 cm diameter for males and 1.5 cm for females), which was positioned horizontally 50 cm above a foam pad. The rat was placed at the center of the beam, and the time to falling off was recorded to a maximum of 60 s.

### Grip strength test

A 0.6 cm diameter rough PVC cord was placed horizontally 50 cm above a foam pad. A rat’s forepaws were placed on the cord, and the animal was then released. The rat was allowed to hang onto the rope for up to 60 s. The test was performed in 2–3 subsequent trials separated by a short break. The longest hanging time obtained during these trials was used for data analysis.

### Inclined plate test

The inclined plate was comprised of a 28 × 30 cm floor with its upper part covered by a 1-mm-thick soft 20 × 30 cm pad and 20-cm-high walls on three sides. The rat was placed at the highest position of the floor, and the maximum inclination angle of the plate on which the rat maintained its body position for 5 s without falling was determined.

### Acute administration of ethanol

As mentioned above, the open field, balance, coordination and muscle-strength tests were repeated after acute i.p. administration of 16% ethanol. The dose of 0.8 g/kg was used for male rats and 1.0 g/kg was used for female rats due to a lower sensitivity to the sedative/hypnotic effects of ethanol in female rats. Ethanol was administered immediately before the open-field test and 10 min before the other tests. Blood was sampled for blood alcohol level determination 30, 60, and 120 min after ethanol administration. For this purpose, the tip of the tail was pinched with the needle and a small volume of blood (i.e., at least 0.05 ml) was collected into heparinized Eppendorf tubes. Ethanol content was measured using the NAD-ADH enzyme spectrophotometric method (Sigma Chemical Co., St. Louis, MO).

### Statistical analysis of the behavioral data

One-way ANOVA was used for analysis of the raw and normalized data derived from behavioral tests. Whenever significant differences were found, post hoc Student Newman Keul’s tests were performed. The chosen level of significance was *p* < 0.05. To determine (ethanol-associated) effect sizes, post-exposure data from each animal was normalized to its own baseline measurement. Weight of the animals did not correlate with the effect size and thus, was not considered as a weight in the normalization. Statistical outliers were identified using modified Thompson Tau and Tukey’s tests and were excluded. In order to assess the impact of exclusion of any partially non-independent data on the results, jackknife sensitivity analyses were conducted iteratively. None of the excluded outliers significantly affected the statistics.

For analysis of the ladder walk video recordings, we estimated the ethanol-induced relative risk (*RR*) of slippage. Hypothesis testing has often been overemphasized and this has led to a negligence of more appropriate approaches such as confidence intervals for interpreting results^68^. We use here the RR as a highly plausible measure of the risk of a certain event happening in one group compared to the risk of the same event happening in another group. We define the relative risk as the ratio of the probability of the ethanol-induced slippage in HOM and WT control animals, i.e., *RR* = *p*_HOM_*(ETH/BL)/ p*_WT_*(ETH/BL)*. Hereby, *ETH* and *BL* denote the distance to first slip after ethanol administration and under baseline conditions, respectively. The confidence interval of 95% was in turn calculated as [*x*_m_-z*σ/√n; *x*_m_ + z*σ/√n], with *x*_m_ representing the ratio of population means, σ the associated standard deviation derived from the Taylor expansion of the variance of the ratio, *n* sample size and z* = *1.96*.

### Ex vivo nerve-muscle experiments

The experiments were performed in 6-month-old male rats and six animals per genotype. The animals were anesthetized with CO_2_ followed by cervical dislocation. The peroneal nerve, together with the extensor digitorum longus (EDL) from both legs, were excised as described previously^69^. Briefly, the neuromuscular tissues were excised and kept in carbogen-bubbled iced cold high-magnesium Ringer solution, as follows: in mM; 128 NaCl, 1.9 KCl, 1.2 KH_2_PO_4_, 26 NaHCO_3_, 0.85 CaCl_2_, 6.5 MgSO_4_, and 10 glucose at pH 7.4. The muscle activity induced by electrical stimulation of the nerve was recorded at constant perfusion of carbogen-bubbled room temperature Ringer–Krebs–Hesenleit solution, as follows: 140 NaCl, 5 KCl, 1.2 KH_2_PO_4_, 2 CaCl_2_, 1.2 MgCl_2_, 20 HEPES, and 6 glucose at pH 7.4. The peroneal nerve was immersed in mineral oil and electrically stimulated using a Grass SD-9 stimulator via a pair of custom-made platinum electrodes. The distal tendon was fixed in the force transducer (AD Instruments MLT1030/D), while the proximal one was fixed in the fixed arm of the force setup. The signal was A/D converted using PowerLab 4/35 and recorded in a computer using LabChart – Pro V8.0. After determining the optimal length of the muscle and the optimal stimulation voltage, the nerve was stimulated during 10 ms for the following protocols. Force-Frequency in response to 1, 5, 10, 15, 20, 25, 30, 35, 40, 45, 50, and 60 Hz electrical stimulation was measured for 500 ms every 3 min. Fatigue in response to 3 Hz continuously for 5 min and fatigue in response to 60 Hz for 500 ms were measured every 3 s, during 5 min. In between each protocol the tissues were rested for 5 min. At the end, the distance between the muscle and the proximal platinum electrode was measured, as well as the muscle length at the optimal length and the muscle mass. The force-frequency data were analyzed by the cross-sectional normalized force, while the force recorded from both fatigue (i.e., the reduction of force production during muscle stimulation) protocols were normalized by the first contraction and analyzed every 30 s. Statistical analyses of the data were performed accordingly to the data set on Graphpad Prism 5.0.

## Electronic supplementary material

Supplementary Information

## Data Availability

All relevant data are available from the authors. All in vivo and ex vivo data have been made available online through Dryad repository^70^. Due to the massive size of molecular dynamics simulations and electrophysiological recordings, the data are safely stored on institutional servers of the authors. Access to the data will be granted to interested readers on demand.
