# Supplementary material for: Alcohol reduces muscle fatigue through atomistic interactions with nicotinic receptors
Source: Commun Biol. 2018 Oct 3;1:159. doi: 10.1038/s42003-018-0157-9 (PMC6170420; doi:10.1038/s42003-018-0157-9)
Supplement: Supplementary file 1 — Supplementary Information [file 42003_2018_157_MOESM1_ESM.pdf]

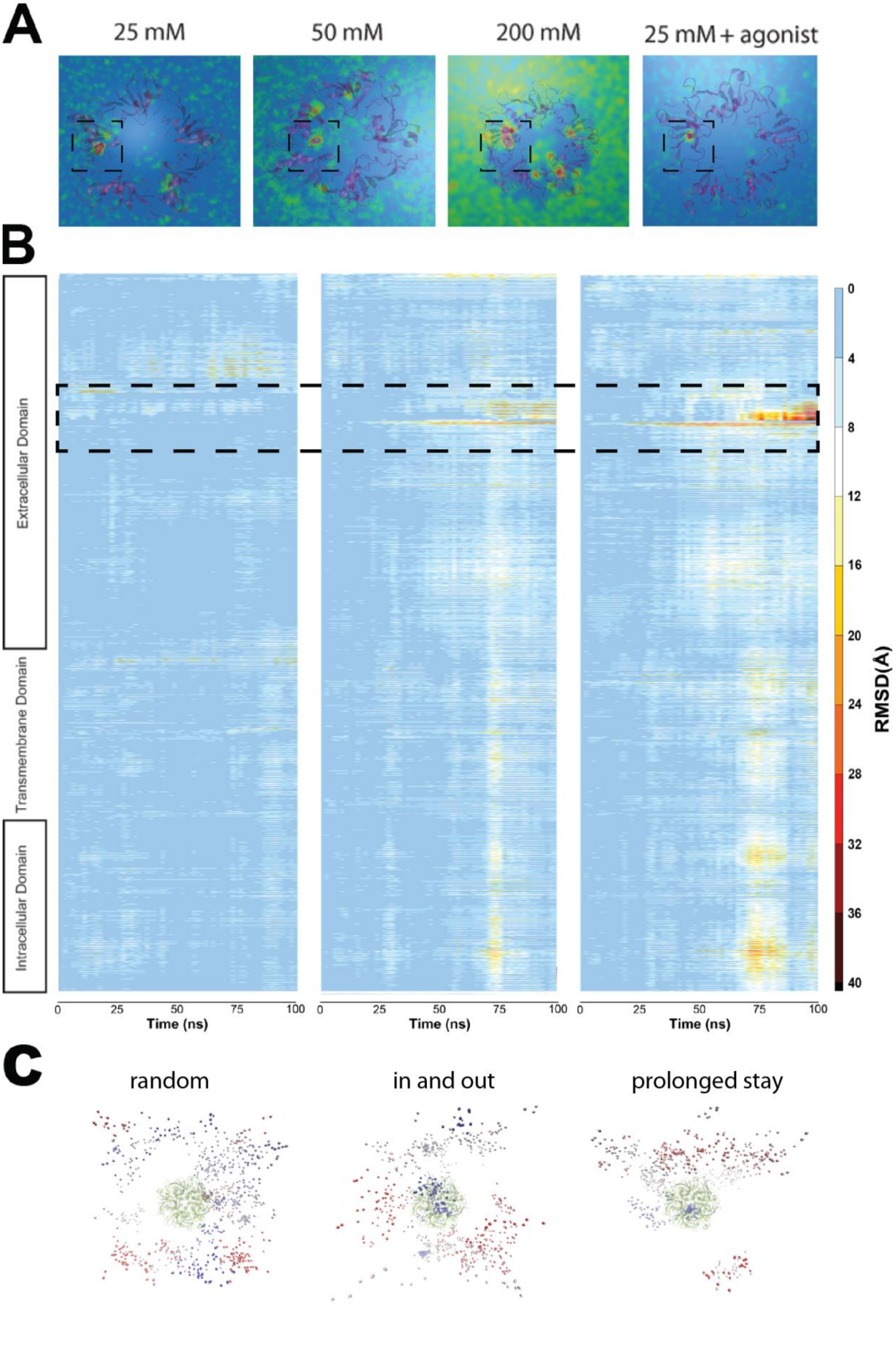
A
25 mM
50 mM
200 mM
25 mM + agonist
B
Extracellular Domain
Transmembrane Domain
Intracellular Domain
0
4
8
12
16
20
24
28
32
36
40
RMSD(Å)
0 25 50 75 100
Time (ns)
C
random
in and out
prolonged stay

Supplementary Figure 1. MD simulations suggest that ethanol interactions with WT of neuromuscular nAChR are largely independent of concentration. (A) Average density distribution of ethanol molecules surrounding the WT receptor for low (25 mM), moderate (50 mM) and high (200 mM) as well as in presence of agonist (acetylcholine) are illustrated. Ethanol molecules occupy with a higher probability the extracellular domain of the α-subunit (area of highest density is shown in red). At high concentrations the density maps further imply a strong stochastic element in the simulations as with higher particle numbers the probability of interactions with other subunits with similar or identical motifs may increase. In addition, the presence of acetylcholine does not affect the probability of ethanol occupancy in the proximity of D83. (B) Absolute value of differences in all-residue heatmaps (HM) between the simulated concentrations (left: HM_{50mM}-HM_{20mM}), (middle: HM_{200mM}-HM_{50mM}) and (right: HM_{200mM}-HM_{20mM}) are presented. The conformational fluctuations induced by simulated ethanol concentrations differ only within a restricted area close to D83 of both α-subunits. (C) While the density distribution maps are suggestive, they represent averages over time and it remains to show what the higher values present. The analysis of the trajectories of each ethanol molecule shows that the majority of molecules move in a Brownian manner and do not interact with the receptor that generate the almost homogenous distribution outside the receptor. The areas of high density are predominantly contributed by particles that accommodate the space close to D83 until the end of simulation, as well as (to a minor degree) by ethanol molecules that move in and out to the site. In this illustration, the beginning of the trajectory is in red, the middle in white, and the end in blue.

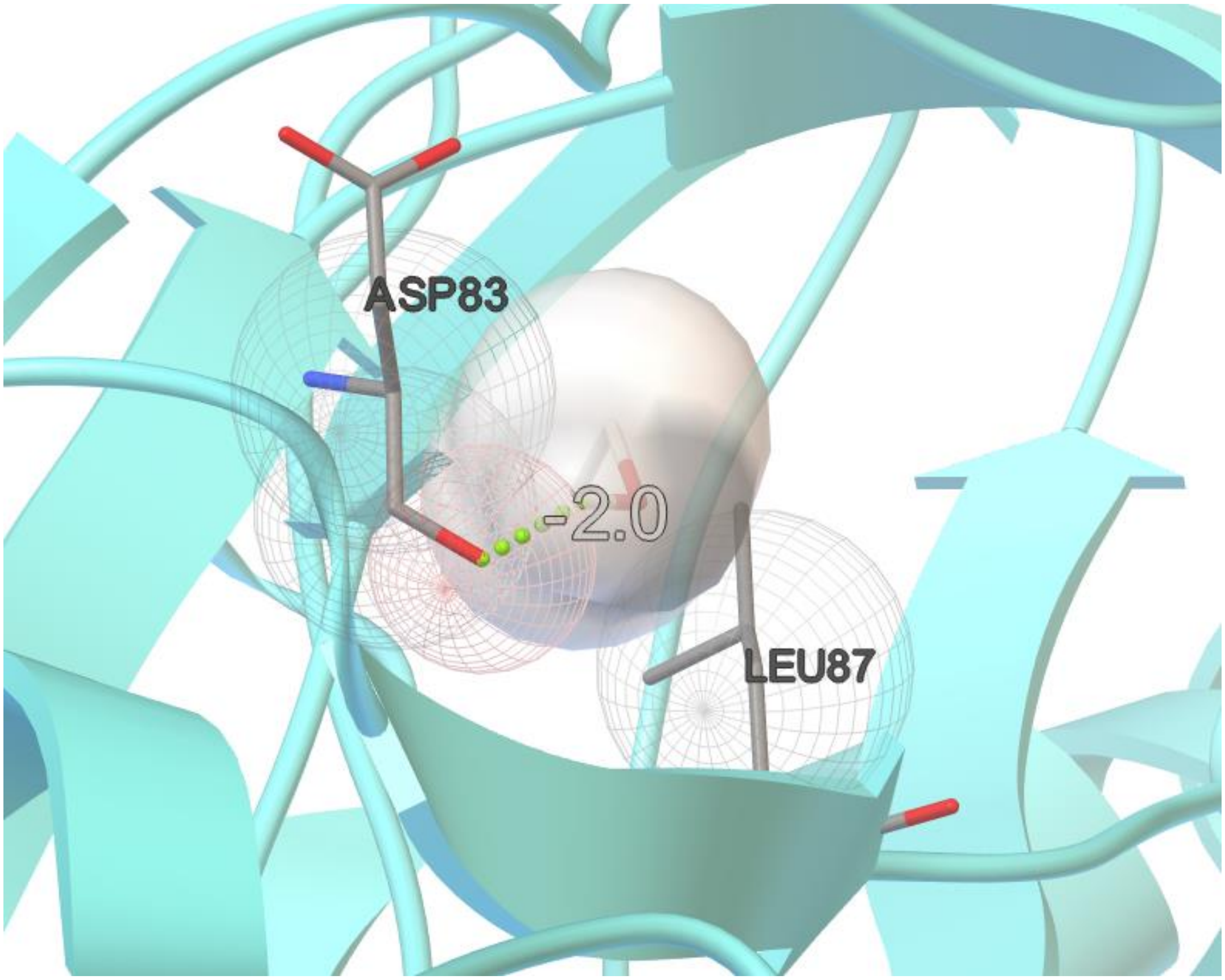

Supplementary Figure 2. Interaction site of ethanol at with aspartic acid (ASP) at α83. Molecular docking suggests a hydrogen bond of -2.0 kcal/mol between ethanol and pore-residing negatively charged amino acid αD83. Ethanol interacts with the backbone carbonyl oxygen and not with the carboxylate group. The oxygens from carboxylate influence the third oxygen from the backbone to organize the necessary electron density for building a hydrogen bond with ethanol

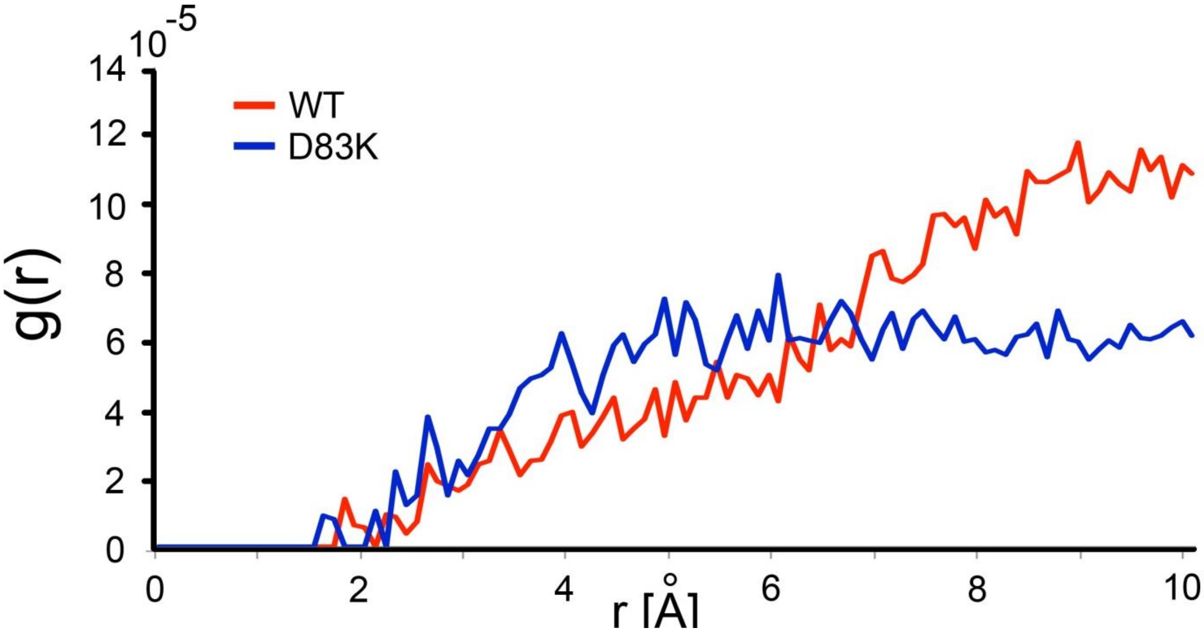

Supplementary Figure 3. Spatial distribution density of ethanol in vicinity of the binding cavity. Radial distribution function *g(r)* shows the lack of selectivity of ethanol towards α83K (red) in contrast to the WT α83D in distances r larger than the interaction cutoff of the simulations (r > 9 Å).

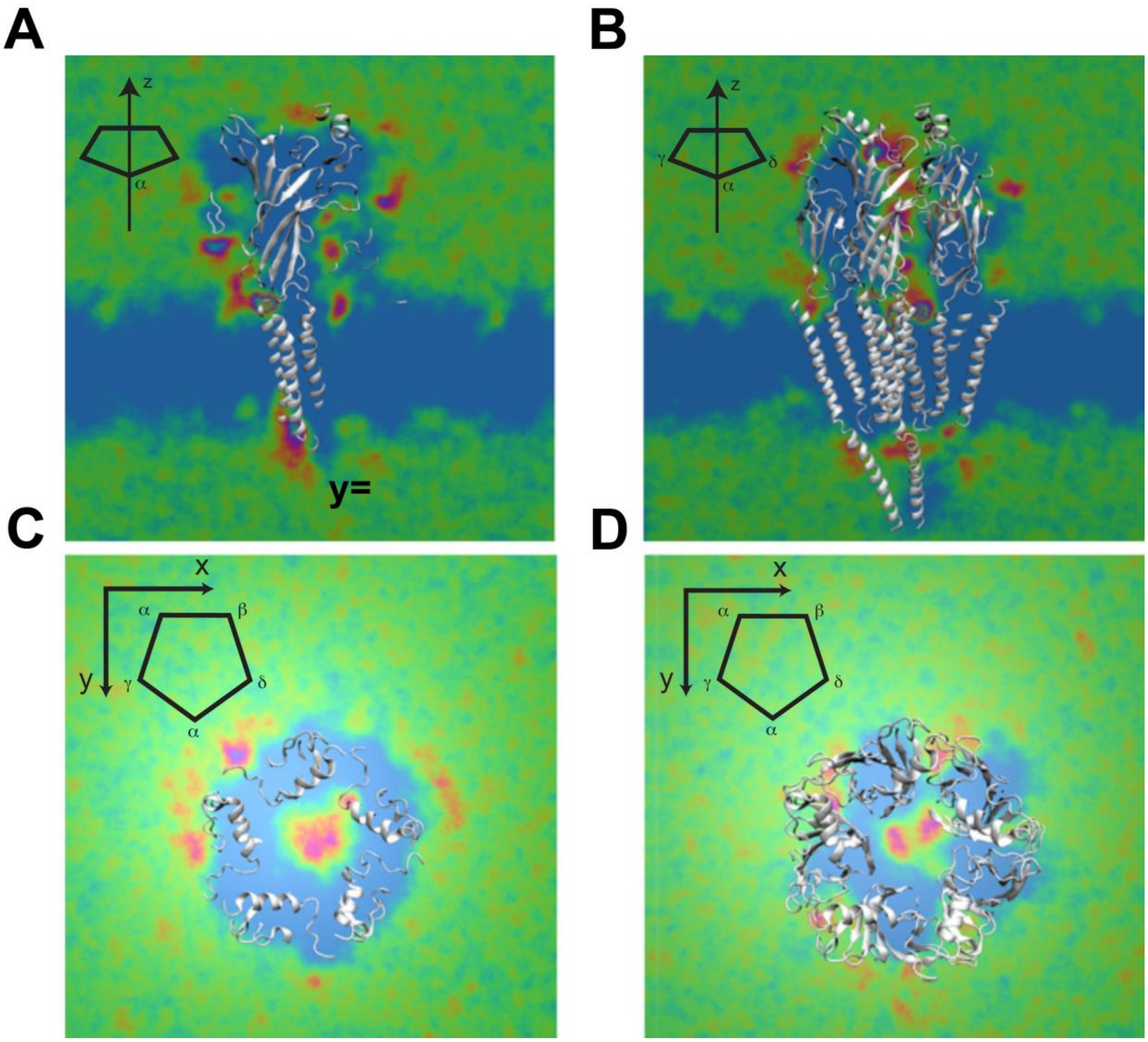

Supplementary Figure 4. Graphical representation of the spatial density distribution (from blue representing the lowest and red the highest density) of acetylcholine molecules around the nicotinic receptor averaged over the 100 ns simulation time. As suggested by experiments, the figures (A,B: surfaces along y-axis; C,D: surfaces along z-axis inwardly oriented) show the enhanced density (red to violet clouds) of the acetylcholine molecules around the α-subunit (A,B), especially in the extracellular and transmembrane domains. Acetylcholine molecules preferably occupy the ligand-binding sites at the α-γ and α-δ interface areas (narrow spaces left and right to α-subunit, respectively). The origin of the coordinate system is located at the lower left corner of the simulation box.

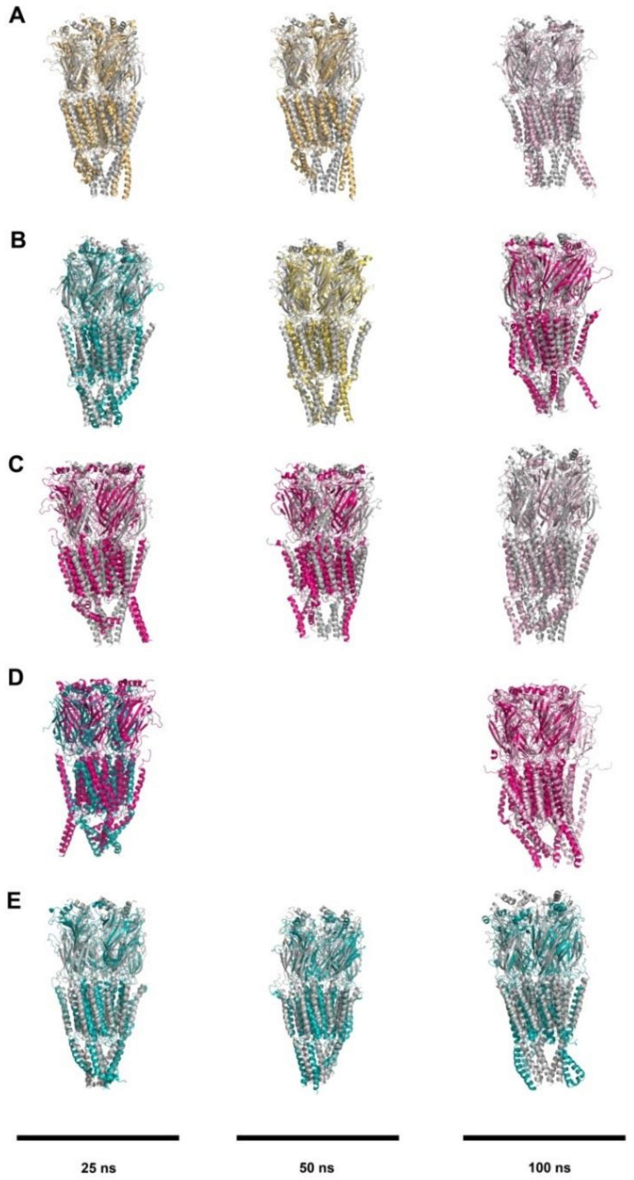

Supplementary Figure 5. Comparative representation of the conformational dynamics of the native and mutant nAChR. A) The conformational changes of the native nAChR in presence of ethanol. B) The effects induced by acetylcholine alone. C) The combined effect of acetylcholine and ethanol molecules. D) The comparison of the effects of acetylcholine and acetylcholine/ethanol effects. E) Dynamics of ethanol-induced effects on the D83K-mutant receptor. As discussed in the main text, despite the presence of alcohol, which accelerates the conformational changes of the receptor (D), the D83K-mutant's conformational variations (inward or outward) appear to not follow the same accelerated time course. Furthermore, the presence of ethanol induces a large outward widening of the intracellular domain throughout the entire simulation period (A and C), an effect that is not present in the mutant (E: 50 ns). Green: closed; Yellow: rotated; Light pink: small pore size fluctuations; pink: moderate pore size fluctuations.

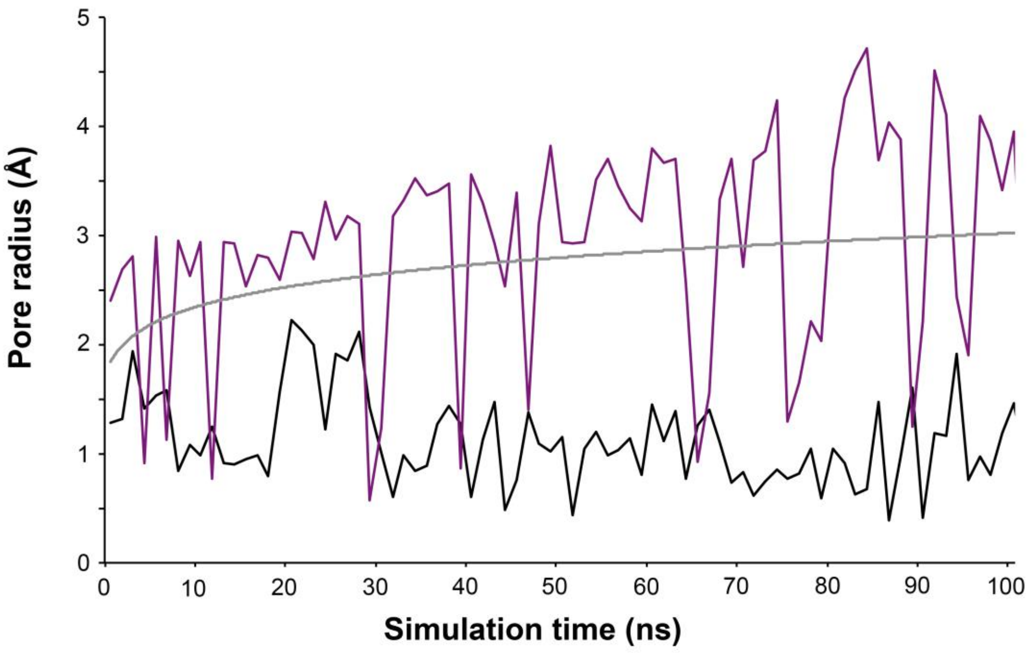

Supplementary Figure 6. Dynamical changes in pore diameter. Time evolution of the pore diameter at the barrier (girdle) suggests a robust closed, non-conductive state (black) in the absence of agonists, whereas ACh (blue) induces a gradual increase in the girdle diameter.

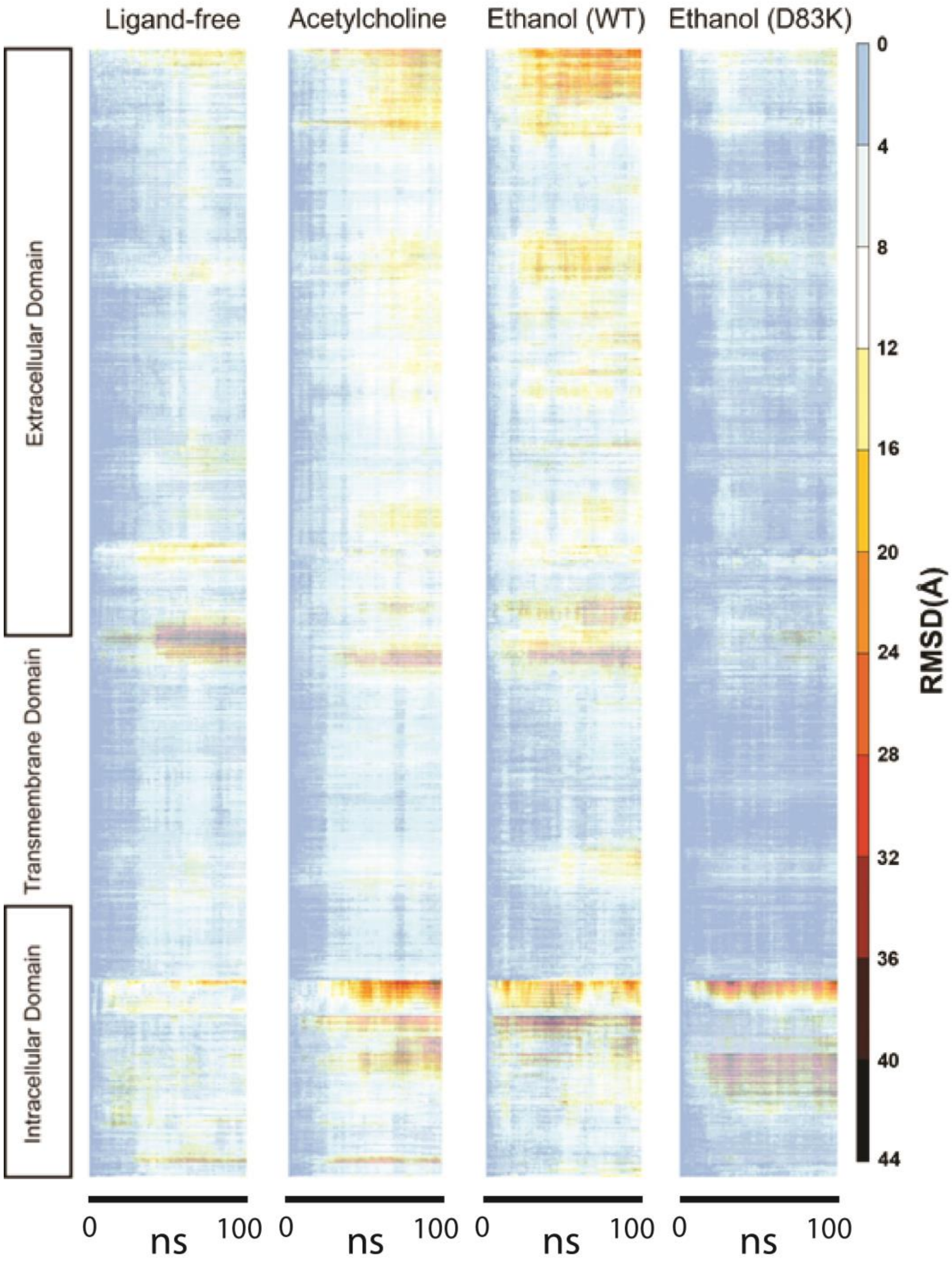

Supplementary Figure 7. Comparison of all-residue heatmaps in the time course of 100 ns (horizontal line). In absence of ligands, the analysis shows variations up to 40 Å (dark red) in the extracellular (α172 and α173) and intracellular domains (α360-α375) and the interface of extracellular and transmembrane domains (α197-α207), which are associated with outward-tilting of the residues due to dynamical fluctuations of the N-terminus and do not represent conformational changes of the receptor. In the presence of ACh molecules, the analysis shows variations up to 40 Å (dark red) in the intracellular domains (α350-α385), transmembrane domain (energy barrier: α251-α256) and the interface of extracellular and transmembrane domains (α185-α210). In the presence of ethanol, the analysis shows similar (with respect

to acetylcholine) variations up to 40 Å (dark red) in the intracellular domains (α350-α385) and the interface of extracellular and transmembrane domains (α185-α210). Furthermore, an increase of the RMSD in the extracellular domain (α65-α90) and in C-terminus is observed. In contrast to the native receptor simulations, the extracellular and transmembrane domains of D83K remain inactive during the entire observation time, although the intracellular alpha helices are in an almost constant state of motion. The heat map further suggests that the changes of the intracellular conformations do not affect the other domains.

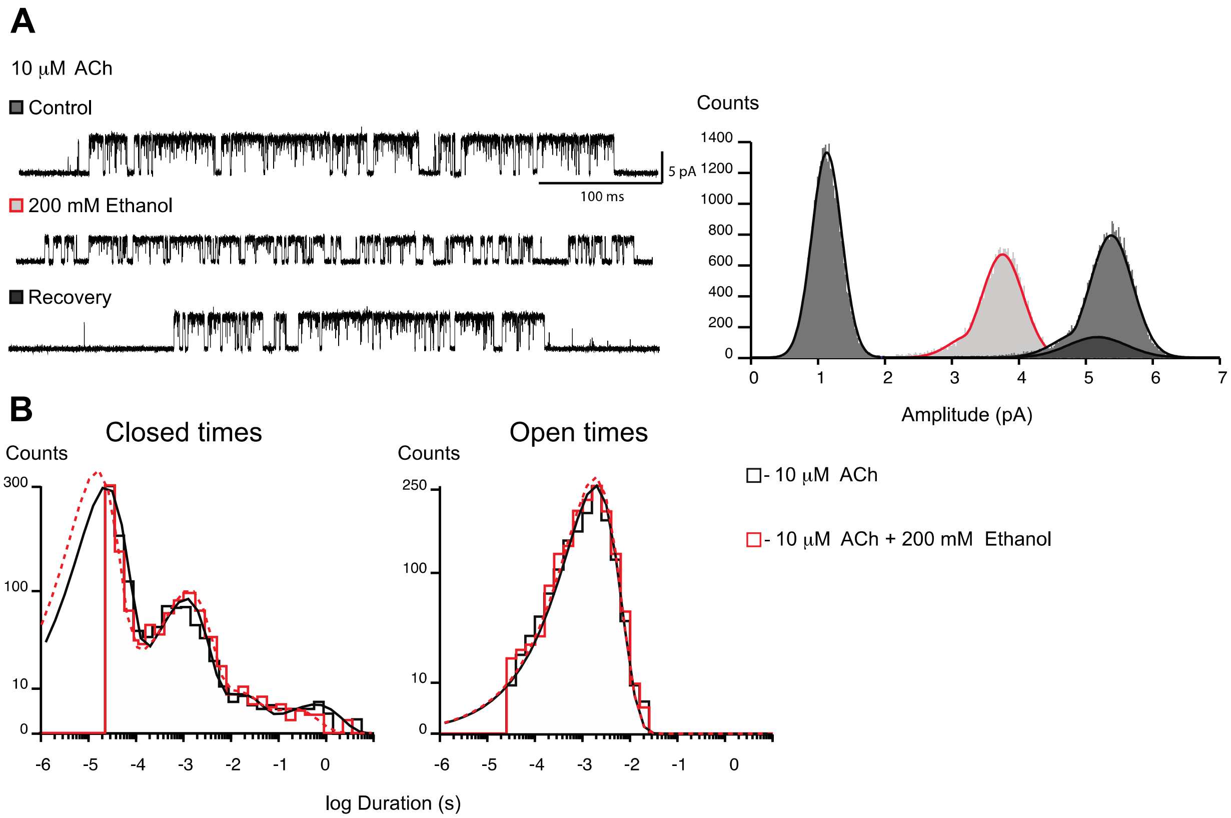

Supplementary Figure 8. Reversal of the reduction of the unitary current amplitude by ethanol and absence of an effect of ethanol on the kinetics of ACh-elicited channel opening and closing. (A). Left, raw current traces recorded from a cell-attached patch at a potential of -70 mV under control conditions (top trace), in the presence of 200 mM ethanol (middle trace), and following removal of ethanol and washing with control solution (lower trace). Right, histogram of digitally sampled points from the raw current traces (20 µs sample interval). The left-most peak corresponds to the baseline current level, whereas the peaks to the right correspond to the open channel current under the three conditions. (B). Histograms of closed and open dwell times from recordings in the presence of 10 µM ACh in the absence (black histogram) or presence of 200 mM ethanol (red histogram). For each histogram, the smooth curves are fits of the sum of exponentials to the data.

A

B

5‘ ATGGAATCCAGATGACTATGGAGGAGTGAAAAAAAATTCACATCCCCTCGGAAAAGATCTGGCGGCCGGGACGTTGTTCTCTATAACAAGTAAGCAAAACCATGC 3‘
5‘ ATGGAATCCAGATGACTATGGAGGAGTGAAAAAAAATTCACATCCCTTCGAAAAAGATCTGGCGGCCGGACGTTGTTCTCTATAACAAGTAAGCAAAACCATGC 3‘

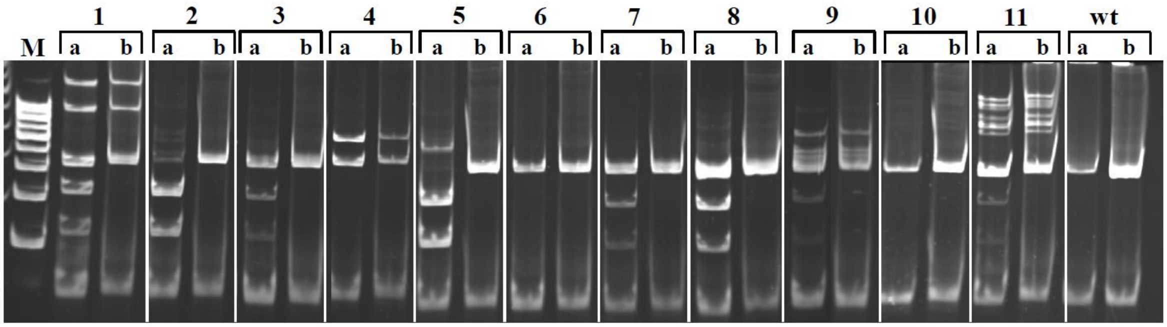

C

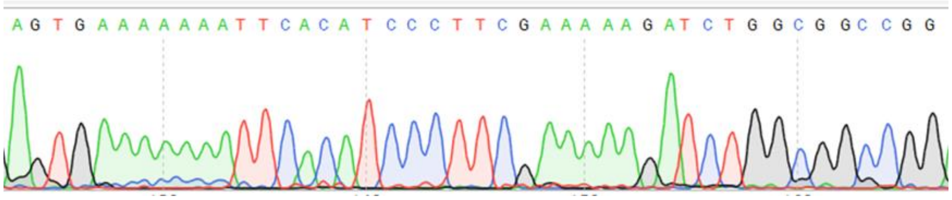

D

guide sequence : CGGCCGCCAGATCTTTTCCG **AGG**
off-target 1: AGGCCGCCAAATCTTTTCAG **GGA**
off-target 2 (exon): T TGCCGACAGATCTTATCCG **AGG**

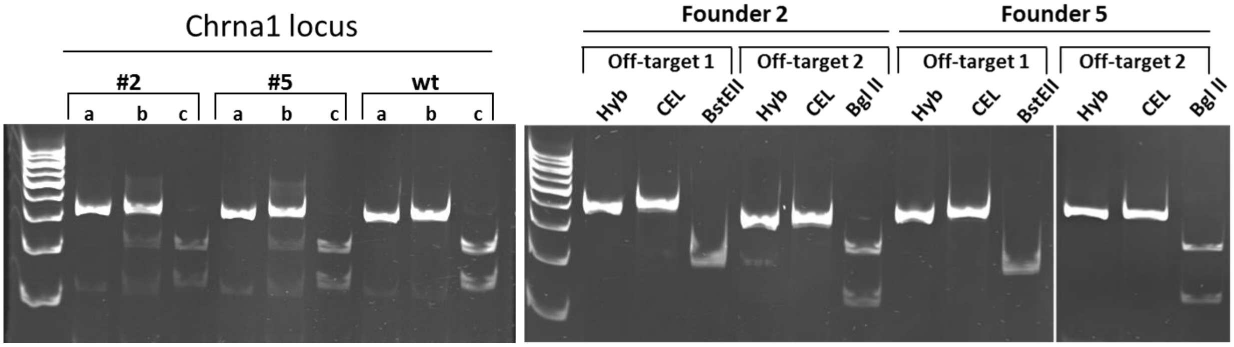

Supplementary Figure 9. Generation of mutant transgenic rats. A) Sequence of DNA donor oligo. The 103 nt donor oligo was designed to replace two nucleotides in CHRNA1 exon 4 and contains 45 and 53 nt homologies flanking the mutations. The wt genomic sequence is displayed on top. The G > A mutation leads to the E > K amino acid exchange. The silent C > T mutation creates a new BstBI restriction enzyme recognition site (TTCGAA) and at the same time destroys the PAM sequence of gRNA target site

(underlined in the wt sequence). B) Restriction digest analysis of 330 bp PCR amplicons spanning the Chrna1 CRISPR/Cas9 target site from eleven founder rats (PAA gel). M, 100 bp DNA ladder (Thermo Scientific), a = BstBI digested (size of digestion fragments 113 + 217 bp), b = undigested. BstBI positive animals indicate oligonucleotide facilitated homologous recombination at the Chrna1 locus. C) Sequence verification of Chrna1 mutant rats. Target locus PCR products were cloned and sequenced. Sequence across the targeting region confirmed correct introduction of desired mutations. D) Off-target analysis. Upper: The sequences of the Chrna1 gRNA target site and the potential off-target sites are shown. gRNA sequence differences are marked in red, the PAM sequence in bold. Lower left: Genomic DNA extracted from founder 2 and 5 was subjected to PCR with on-target primers and subsequent Surveyor nuclease digestion (CEL). PCR products are shown undigested (a) or after CEL digestion (b). As control the fragment is cut with the restriction enzyme Bgl II (c) present also in the wt sequence. Appearing fragments in CEL digested samples indicate genomic mutations. Lower right: Genomic founder DNA was amplified with off-target primer and analyzed using the Surveyor nuclease assay. No digestion products are observed after CEL digestion. As positive control the PCR fragment is cut with an appropriate restriction enzyme as indicated.

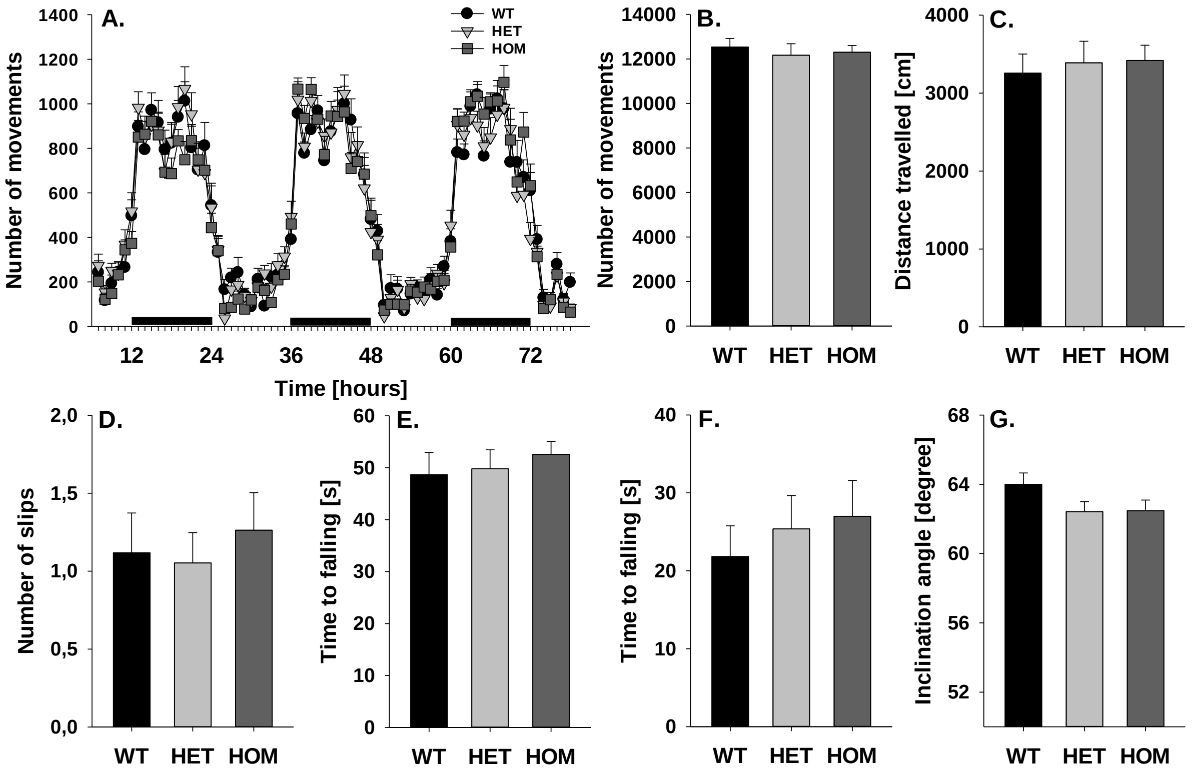

Supplementary Figure 10. Locomotor activity, motor coordination, balance and grip strength measured under the baseline conditions in wild-type (WT, n=17), heterozygous (HET, n=19) and homozygous (HOM, n=19) CHRNA1 mutant rats. Locomotor activity measured in the home-cages as (A) number of movements performed every hour during three consecutive days (black horizontal bars mark the dark (active) phases of the circadian cycle) and (B) average number of movements performed during 24 hours, and as (C) total distance travelled in the 30-min open-field test. Motor coordination and balance measured as (D) number of slips in the ladder rung walking test and (E) time to falling in the equilibrium test. Grip strength measured as (F) time to falling in the grip strength test and (G) maximum inclination angle in the inclined plate test. Data are presented as means ± S.E.M.

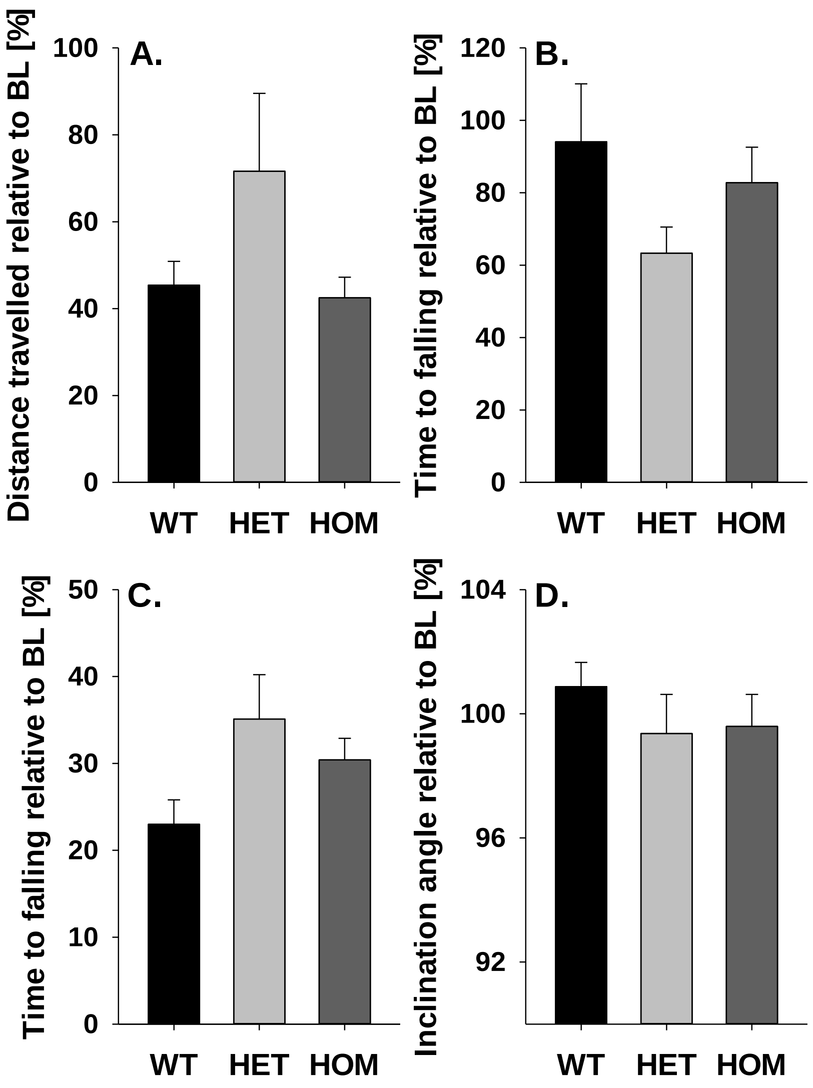

Supplementary Figure 11. Locomotor activity, motor coordination, balance and grip strength measured following acute administration of ethanol in wild-type (WT, n=13), heterozygous (HET, n=14) and homozygous (HOM, n=15) CHRNA1 mutant rats. (A) Locomotor activity measured as total distance travelled in the 30-min open field test. Motor coordination and balance measured as (B) time to falling in the equilibrium test. Grip strength measured as (C) time to falling in the grip strength test and (D) maximum inclination angle in the inclined plate test. Motor coordination, balance and grip strength were measured 10 min after acute administration of ethanol. The data is normalized to baseline (BL) measurements. Data are presented as means ± S.E.M.

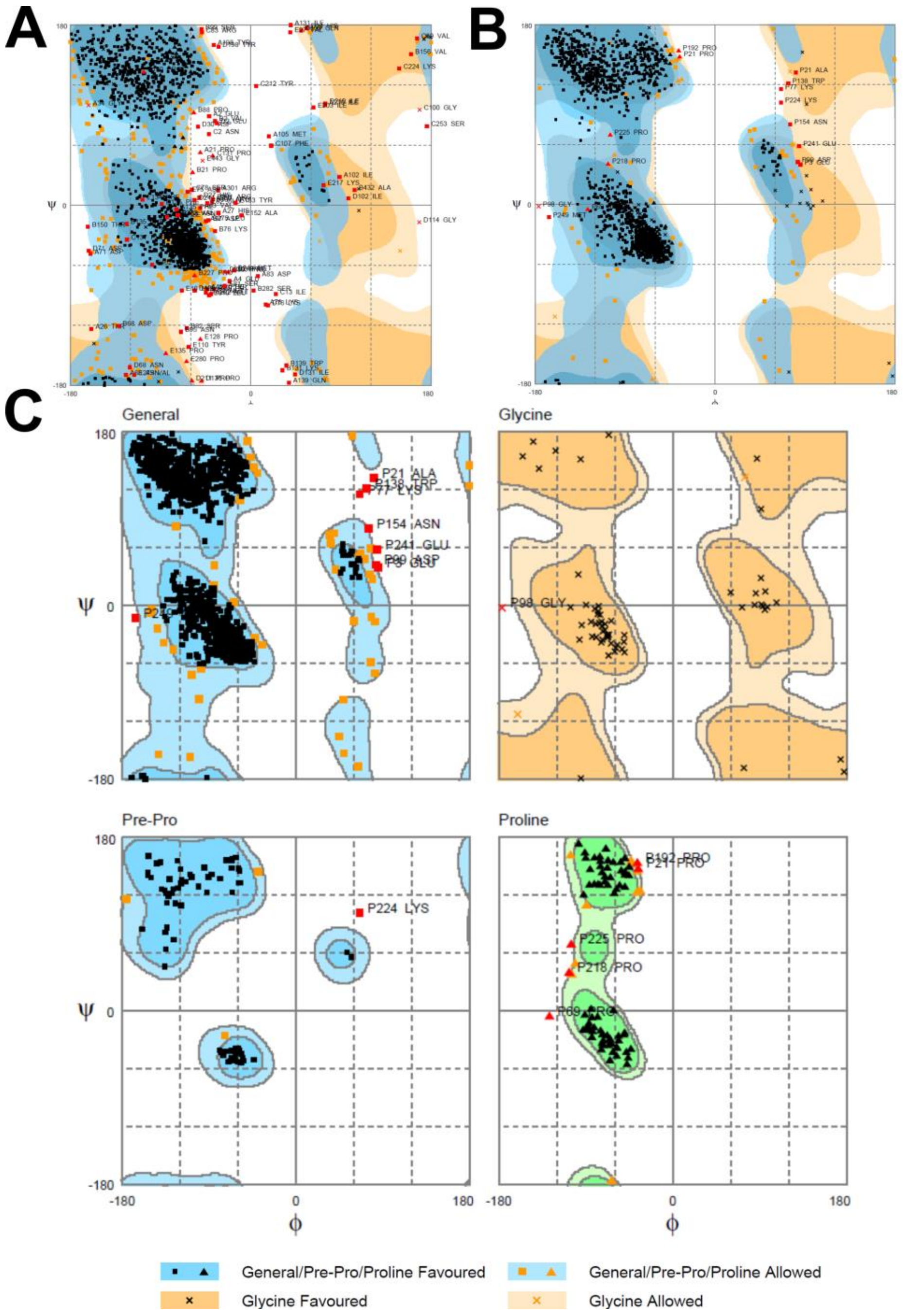

Supplementary Figure 12. Analysis of Ramachandran plot in order to visualize energetically allowed regions for backbone dihedral angles ψ against φ of amino acid residues in Torpedo protein structure. (A) The plot of 2BG9 model shows that number of residues found in the favored region 81.7% from the total residues, and number of residues in allowed region is 12.9% and number of residues in outlier region 5.4%. (B) The starting conformation for MD simulations (ie, after energy minimization) shows clear improvements. The number of residues found in the favored region is 95.6% from the total residues, and number of residues in allowed region is 3.5% and number of residues in outlier region 0.09%. (C) The simulation model shows general, glycine, pre-proline, and proline plots exhibits the possible ψ and φ dihedral angles and allowed and disallowed regions of complete amino acids.

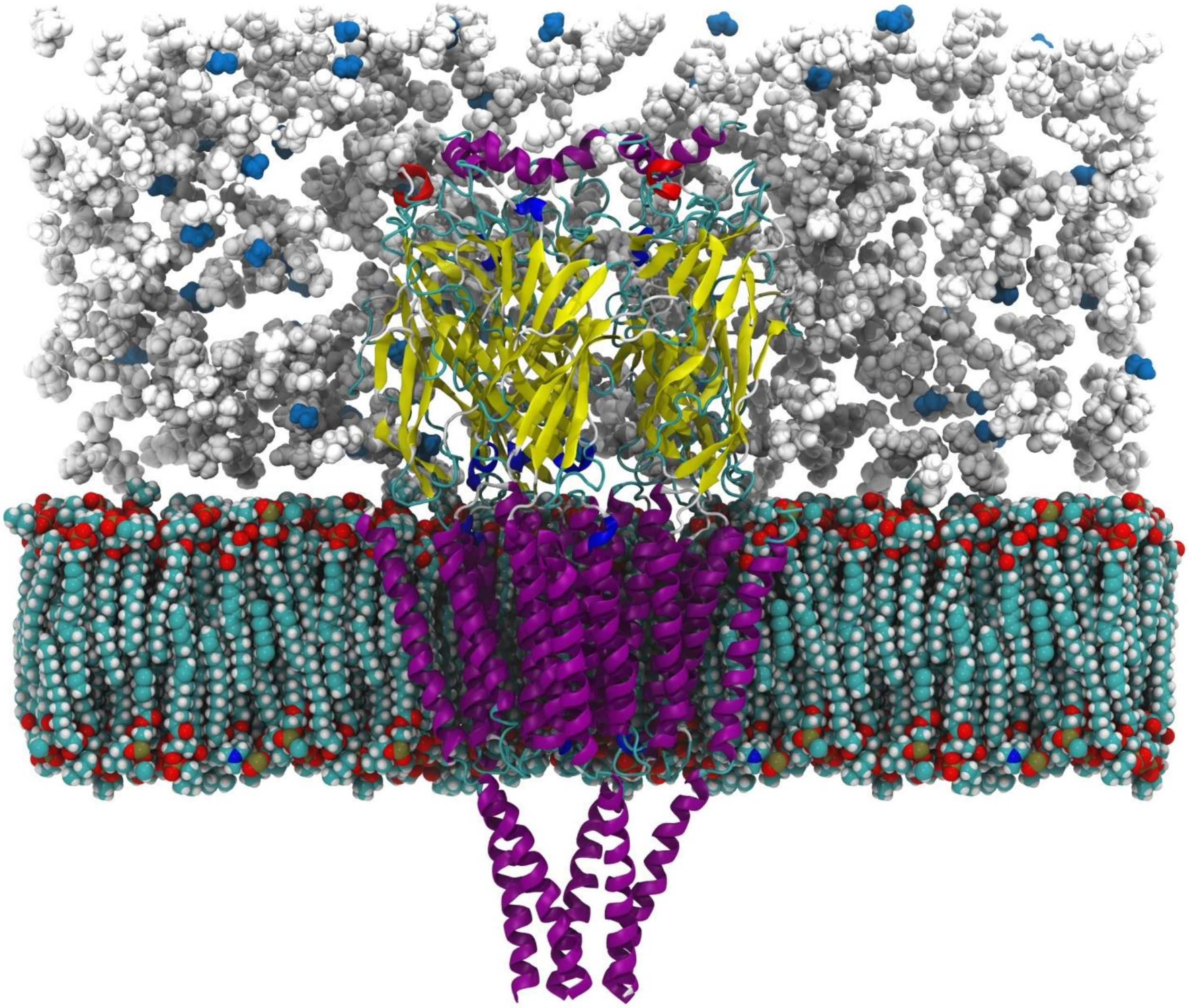

Supplementary Figure 13. Graphical illustration of the general setup of molecular dynamics simulations. The WT nAChR is embedded within the 200 Å x 200 Å POPC membrane. ACh (grey) and EtOH (blue)molecules are distributed randomly within the extracellular space.

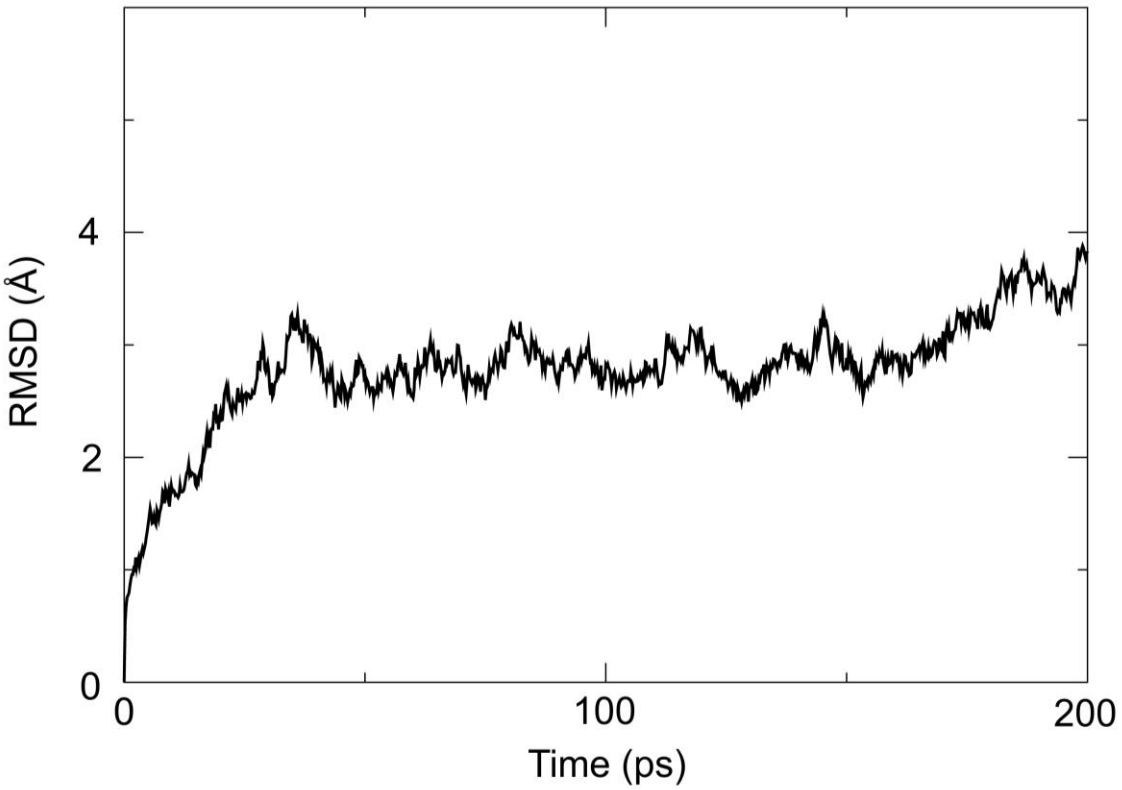

Supplementary Figure 14. Root mean squared deviation of the nAChR during equilibration. RMSD of the simulation system suggests that 200 ps are sufficient for the NpT equilibration of receptor backbone, ethanol and acetylcholine molecules.

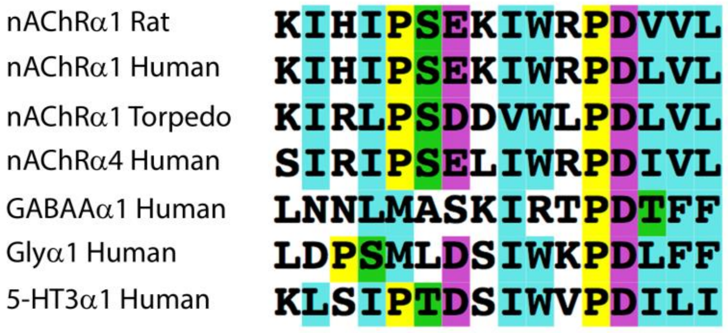

Supplementary Figure 15. Sequence alignment of α-subunits of cys-loop receptors that interact with ethanol. Molecular docking suggests that the interaction in the extracellular domain involves α83. Sequence alignment of the attractive extracellular α-subunit cavity among different species (rat, human and torpedo) and between other Cys-loop receptors known to interact with ethanol (purple: negative charge; yellow: prolines; green: polar; cyan: aromatic residues).

	ASP (WT)	GLU	LYS	GLY	SER
**Area under the curve**	5.4 x10^{-4}	2.9 x10^{-4}	4.6 x10^{-4}	7.7 x10^{-5}	3.2 x10^{-4}
**Initial SASA [Å^2]**	2142.63	2331.99	2253.96	1971.47	2041.85
**Average SASA [Å^2]**	2174.73	2363.32	2307.47	1948.40	2076.80

Supplementary Table 1. Computational estimates of changes in solvent accessible areas of binding cavities for different amino acid substitutions. The area under the curves of the calculated *g(r)* functions of the wild type and mutant receptors, as well as the initial and average solvent accessible surface areas. Hereby, the initial and average solvent accessible surface areas did not differ significantly.

	**Day 1**	**Day 2**	**Day 3**	**Day 4**	**Day 5**	**Day 6**	**Day 7**
**Week 1**	Locomotor Activity in Home Cages (3 Days of Continuous Measurement)			Break	Break	Break	Open-Field-Test Habituation (2h)
**Week 2**	Open-Field-Test (30 Minutes)	Break	Break	Break	Break	Break	Coordination Tests
**Week 3**	Break	Break	Break	Injection & Open-field-Test	Break	Break	Break
**Week 4**	Break	Break	Break	Break	Injection, Coordination Tests & Blood Sampling		

Supplementary Table 2. in vivo experiment time-line design. Experiments spread over four weeks. In the first two weeks, baseline locomotor activity in home cages, open-field and coordination tests were conducted while WT, HET and HOM animals received a saline injection. In order to avoid any learning effects, tests with alcohol injection were conducted at least with 10 days after the baseline measurement. Considering the pharmacokinetics of ethanol in rats, a break of 7 days between the two ethanol injections was chosen. Each animal went through the experiment once. Subsequently, animals were sacrificed to extract the EDL muscle for ex vivo investigations.
